# Mechanotransduction through oxidative stress upregulates circLyst to drive vascular remodeling in hypertensive humans and rodents

**DOI:** 10.1186/s43556-026-00593-3

**Published:** 2026-10-13

**Authors:** Juanjuan Tan, Weiyi Hu, Li Shen, Biyang Luo, Huajin Liu, Feng Li, Hui Qin, Yuanwang Yu, Haifang Wang, Jing Dong, Qi Zhang, Hong Zhang, Fan Lv, Yingxin Qi, Kai Wang, Yi Yang, Zhiqiang Yan, Kai Huang

**Affiliations:** 1 Shaanxi University of Chinese Medicine, Xianyang, 712046 China; 2 Institute of Mechanobiology & Medical Engineering, School of Life Sciences & Biotechnology, Shanghai Jiao Tong University, 800 Dongchuan Road, Minhang, Shanghai, 200240 China; 3 The Third Hospital of Changha (Changsha Hospital Affiliated to Hunan University), Changsha City, 410015 Hunan Province China; 4 Shanghai Jiao Tong University Affiliated Sixth People’s Hospital South Campus, Shanghai, China; 5 Department of Pediatric Surgery, Xinhua Hospital, Shanghai Jiao Tong University School of Medicine, 1665 Kongjiang Road, Yangpu, Shanghai, 200092 China; 6 Department of Cardiovascular Surgery, Ruijin Hospital, Shanghai Jiao Tong University School of Medicine, Shanghai, 200025 China; 7 The Key Laboratory of Bioactive Materials, Ministry of Education, College of Life Sciences, Nankai University, Tianjin, 300071 China; 8 Key Laboratory for the Genetics of Developmental and Neuropsychiatric Disorders, Ministry of Education, Shanghai Jiao Tong University, 800 Dongchuan Road, Minhang, Shanghai, 200240 China

**Keywords:** Mechanical stress, Oxidative stress, Circ_2465 (circLyst), CeRNA, Vascular remodeling, Hypertension

## Abstract

Aberrant cyclic stretch serves as a critical driver of vascular remodeling in hypertension, yet the underlying molecular mechanisms remain largely unknown. Here, we studied the role of a novel circRNA, termed circ_2465 (circLyst), in vascular remodeling in both human and rodent samples under high cyclic stretch. Next-generation sequencing data revealed that 165 circRNAs were differentially expressed in the thoracic aortas of spontaneously hypertensive rats (SHRs) compared with normotensive Wistar–Kyoto rats (WKYs), of which 60 circRNAs were significantly upregulated while 105 circRNAs were significantly downregulated. CircLyst, verified as a novel circRNA by ribonuclease R (RNase R) digestion and Actinomycin D assays, was induced in the thoracic aortas of SHRs and stably located in the cytoplasm as detected by fluorescence in situ hybridization (FISH). This elevation was confirmed in hypertensive patients and was recapitulated in vascular smooth muscle cells (VSMCs) exposed to high cyclic stretch in vitro. Mechanistically, oxidative stress, mediated by NADPH oxidases NOX1 and NOX4, induced circLyst expression. CircLyst then functioned as a sponge for miR-298-5p, derepressing its target gene Col23a1, thereby promoting VSMC proliferation and migration—key events in vascular remodeling. Importantly, both systemic and VSMC-specific targeting of circLyst attenuated pathological remodeling and blunted hypertension in animal models. Collectively, our findings establish the oxidative-stress-driven circLyst/miR-298-5p/Col23a1 axis as a critical regulator of vascular remodeling and highlight circLyst as a potential therapeutic target for hypertensive vascular disease.

**Supplementary Information:**

The online version contains supplementary material available at https://doi.org/10.1186/s43556-026-00593-3.

## Introduction

Vascular remodeling, an important and common pathological event in cardiovascular diseases (CVDs), is the process of altering structure and arrangement in blood vessels through cell dysfunctions and production or degradation of the extracellular matrix (ECM) [1–3]. It has been established that vascular remodeling contributes to the development of a variety of vascular diseases including high blood pressure [4–7], atherosclerosis and restenosis [4, 8–10], aortic aneurysms [4, 11–13], neointima formation post vascular injury [14, 15] and vein graft failure [16–18].

Mechanical stress and oxidative stress contribute to cell function, dysfunction, and various processes, including remodeling, development, homeostasis, and aging [19–23]. In the cardiovascular system, mechanical stress primarily acts in three directions: shear stress, hydrostatic pressure, and cyclic stretch [24–26]. Abnormal cyclic stretch induced vascular remodeling in hypertension [5, 27, 28] and grafted vein [16, 29]. The primary role of NADPH oxidase (NOX) is to produce reactive oxygen species (ROS), making it one of the primary enzymes responsible for generating oxidative stress in the cardiovascular system [4]. NOXs derived oxidative stress played important roles in vascular remodeling during the progress of hypertension [4, 30], aortic aneurysms [4, 12, 13, 31] and atherosclerosis [4, 32, 33]. Nevertheless, the role of mechanical stress induced ROS in vascular remodeling of hypertension has remained incompletely understood.

Circular RNAs (circRNAs) are covalently closed single-stranded RNAs (ssRNAs) in eukaryotes with tissue- and cell-specific expression patterns [34, 35]. Most circRNAs are produced with a 3′,5′-phosphodiester bond between the back-spliced exons by a non-canonical splicing event called backsplicing from middle exons of annotated genes [34, 35]. CircRNAs are found to modulate gene expression by regulating transcription and splicing, titrating microRNAs (miRNAs)/acting as miRNA sponges, interacting with proteins and acting as templates for the synthesis of polypeptides by a cap-independent translation manner through internal ribosome entry sites [34, 36]. Given that circRNAs exhibit greater stability than their cognate linear transcripts, possess unique structural conformations, and are often expressed in a disease-specific manner, they have drawn considerable attention in cardiovascular research [36, 37]. Emerging evidence indicates that circRNAs modulate cell death in cardiovascular diseases, contribute to vascular aging and aging-related vascular disorders, and hold promise as mechanistic regulators, diagnostic biomarkers, and therapeutic targets [38–40]. Accordingly, further studies are warranted to fully elucidate their clinical potential as both biomarkers and therapeutic agents in cardiovascular diseases.

In the present study, we aimed to investigate the functional role and molecular mechanisms of circRNA_2465 (circLyst) in vascular remodeling under pathological cyclic stretch, and to determine whether its modulation affects hypertensive vascular remodeling. We further sought to evaluate the potential of circLyst as a therapeutic candidate in this context.

## Results

### Cyclic stretch elevates circLyst in hypertensive humans and rats

**Fig. 1:**
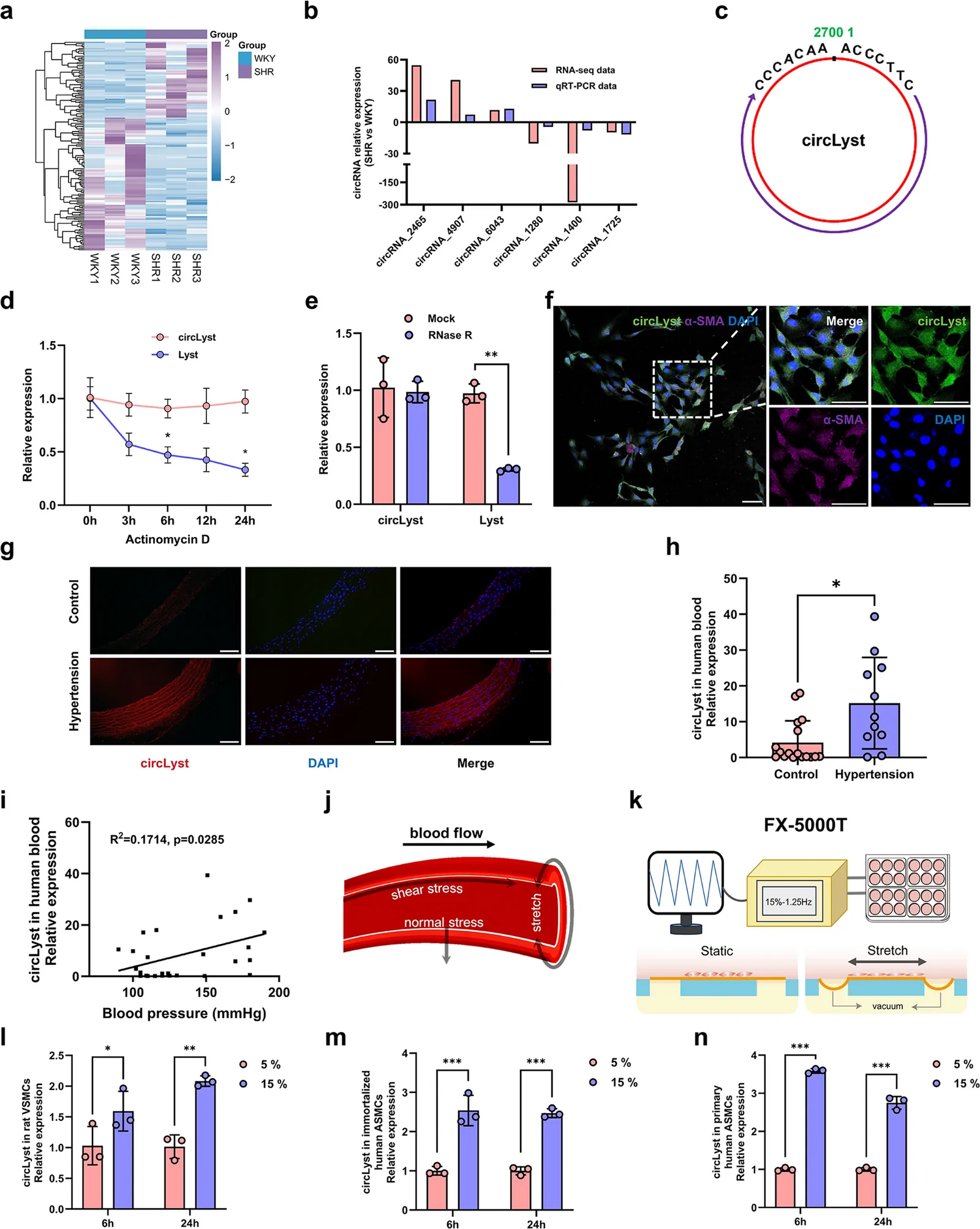
CircLyst was highly expressed in hypertension under cyclic stretch. **a** Heat map of differentially expressed circRNAs in the thoracic aortas of spontaneously hypertensive rats (SHRs) and normotensive Wistar-Kyoto rats (WKYs). **b** The expression of three upregulated and three downregulated circRNAs in the thoracic aortas of SHRs compared with WKYs detected by next-sequencing and verified by qRT-PCR. **c** Schematic diagram of circLyst with the length of 2, 700 bp. **d** qRT-PCR assays were conducted to detect the expressions of circLyst and Lyst mRNA in VSMCs after actinomycin D treatment. The values are expressed by mean ± SD. *n* = 3 at each time point. Two-way ANOVA followed by Bonferroni-corrected Welch T tests: *p = 0.0153 (6 h), *p = 0.0118 (24 h), linear Lyst relative to circLyst at each matched time point. **e** Total RNAs were digested with RNase R followed by qRT-PCR detection of circLyst and Lyst mRNA. Lyst mRNA was detected as the RNase R-sensitive control. The values are expressed by mean ± SD. n = 3, Two-way ANOVA followed by Bonferroni-corrected Welch T tests: **p = 0.0010 (Lyst), RNase R group versus Mock group. **f** Fluorescence in situ hybridization (FISH) was performed to detect circLyst (Cy3-labeled probes) and α-SMA (FAM-labeled probes) in VSMCs. Nuclei were stained with 4ʹ,6-diamidino-2-phenylindole (DAPI), Scale bar = 50 μm. **g** FISH assay was performed to detect circLyst expression in aortas of SHRs and WKYs using Cy3-labeled probes (circLyst). Nuclei were stained with 4ʹ,6-diamidino-2-phenylindole (DAPI), Scale bar = 100 μm.** h** Blood samples were harvested from people with high and normal blood pressure. qRT-PCR was conducted to detect circLyst expression in the blood samples. *n* = 11–17, Welch T test: *p = 0.0193, circLyst relative expression in the Hypertension group versus the Control group.** i** Correlation analysis of blood pressure and circulating circLyst levels indicating that circulating circLyst is positive correlated with blood pressure, R^2^ = 0.1714, *p* = 0.0285. *n* = 28.** j** Schematic diagram of the main source of mechanical stress in vascular system. Shear stress is induced by blood flow. The gravity of the blood generates normal stress (hydrostatic pressure). Cyclic stretch in vessels is produced by the deformation caused by cardiac contraction and relaxation as well as hemodynamic generated by blood flow. **k** Working scheme of applying cyclic stretch on VSMCs with FX5000T Strain Unit, cyclic stretch is induced by the vacuum pump controlled by FX-5000 T system. **l** Rat VSMCs was harvested and subjected to 5% and 15% cyclic stretch respectively, circLyst was detected in VSMCs under cyclic stretch for 6 h and 24 h respectively. *n* = 3, Two-way ANOVA followed by Bonferroni-corrected Welch T tests: *p = 0.0482 (6 h), **p = 0.0015 (24 h), 15% cyclic stretch versus 5% cyclic stretch. **m** Immortalized human ASMCs were subjected to 5% and 15% cyclic stretch respectively, circLyst was detected in ASMCs under cyclic stretch for 6 h and 24 h respectively. *n* = 3, Two-way ANOVA followed by Bonferroni-corrected Welch T tests: ***p < 0.001 (6 h), ***p < 0.001 (24 h), 15% cyclic stretch versus 5% cyclic stretch.** n** Primary human ASMCs were subjected to 5% and 15% cyclic stretch respectively, circLyst was detected in ASMCs under cyclic stretch for 6 h and 24 h respectively. *n* = 3, Two-way ANOVA followed by Bonferroni-corrected Welch T tests: ***p < 0.001 (6 h), ***p < 0.001 (24 h), 15% cyclic strain versus 5% cyclic stretch

To explore the potential role of circRNA in vascular remodeling during hypertension, the next-generation sequencing was applied to the thoracic aortas of SHRs and WKYs. Through sequencing, a total of 165 circRNAs displayed significant differential expression in SHRs compared with WKYs, of which 60 circRNAs were significantly upregulated, and 105 circRNAs were significantly downregulated (Fig. 1a). To validate the sequencing data, three upregulated and three downregulated circRNAs among the differentially expressed circRNAs were selected for qRT-PCR analysis filtered by the fold change, raw intensity and the length of circRNAs. The results showed that rno-circRNA_2465 exhibited the highest upregulation among the analyzed circRNAs (Fig. 1b). It was found to be upregulated by a remarkable 21.65-fold in the thoracic aorta of SHRs compared to WKYs quantified by qRT-PCR (Fig. 1b) and the sequence length of rno-circRNA_2465 was determined to be 2, 700 bp (Fig. 1c and Table S1). According to NCBI BLAST, the sequence of rno-circRNA_2465 was matched with the rattus norvegicus Lyst gene. Therefore, we named rno-circRNA_2465 as circLyst and explored its role in vascular remodeling of hypertension. To compare the stability of circLyst and linear transcripts, rat VSMCs were treated with the transcriptional inhibitor actinomycin D. The expression of circLyst dropped slightly at 24 h (< 20%) while the expression of linear Lyst mRNA markedly declined over time (approximately 70% at 24 h) (Fig. 1d). Besides, the total RNAs extract from VSMCs was treated with RNase R (an exoribonuclease that digests linear RNAs but preserves circRNAs). The reduction of circLyst was also less pronounced than that of Lyst (Fig. 1e). The results indicated that circLyst had a longer half-life and was more stable than Lyst mRNA. Fluorescent in situ hybridization (FISH) assay confirmed robust expression of cytoplasmic circLyst in VSMC (Fig. 1f). In addition, FISH data further verified that circLyst was highly expressed in the cytoplasm of the aorta from SHR compared with the aorta of WKY (Fig. 1g). Then, the expression of circLyst was detected in blood samples of hypertensive and normotensive human. The expression of circLyst was significantly upregulated in hypertension (Fig. 1h). Of note, linear correlation between circLyst expression and blood pressure was calculated. The elevated circLyst levels correlated with increased blood pressure in human (Fig. 1i).

Blood vessels are exposed to mechanical environment due to the contraction and relaxation of the heart, the mechanical forces (Fig. 1j) are mainly in three directions: shear stress, hydrostatic pressure and cyclic stretch [24, 25]. Others and we have shown that abnormal cyclic stretch plays a vital role in vascular remodeling [24, 25, 27, 28, 41]. Therefore, the expression of circLyst in VSMCs was determined subjected to the normal level (5%) of cyclic stretch and a high level (15%) of cyclic stretch using FX-5000 T Strain Unit (Fig. 1k). Compared with 5% cyclic stretch, 15% cyclic stretch increased the mRNA level of circLyst after 24-h application in rat VSMCs (Fig. 1l). The expression of circLyst was also detected in immortalized human ASMCs. CircLyst was increased significantly after 6 h and 24 h of 15% cyclic stretch (Fig. 1m). Similar result was confirmed in primary human ASMCs, where circLyst increased significantly after 6 h and 24 h under the condition of 15% cyclic stretch compared to the 5% cyclic stretch (Fig. 1n).

These findings suggest that circLyst was elevated by high cyclic stretch in hypertension both in human and rat.

### ROS induced by high cyclic stretch promote circLyst expression

**Fig. 2:**
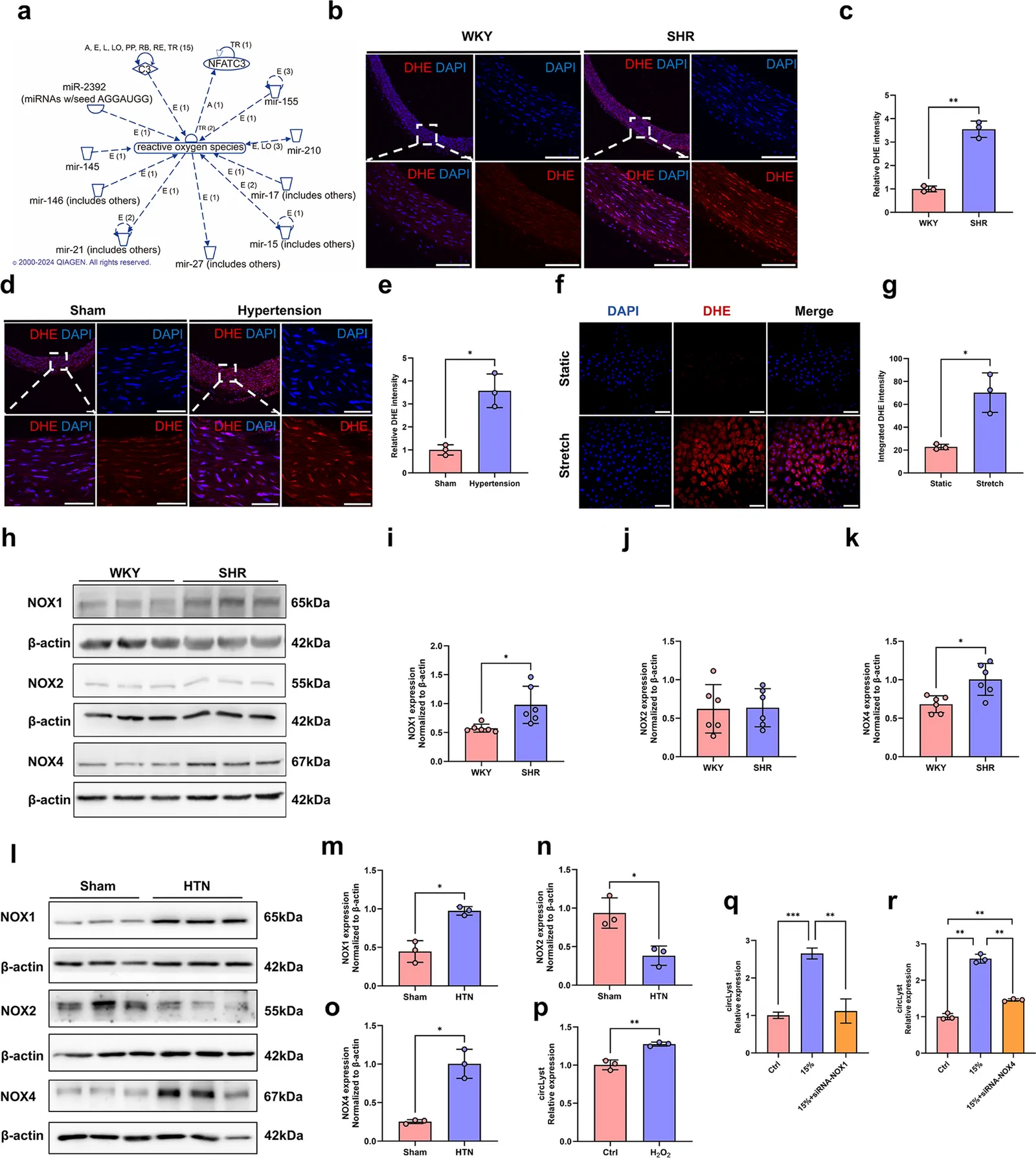
High cyclic stretch induced NOX-ROS signaling promote circLyst expression. **a** Ingenuity Pathway Analysis (IPA) explored the interaction between reactive oxygen species (ROS) and non-coding RNA. **b-c** Aortic production of superoxide in SHRs and WKYs was detected by dihydroethidium (DHE) imaging, Scale bar = 100 μm. *n* = 3, Welch T test: **p = 0.0030, SHR group versus WKY group. **d-e** DHE staining was used to detect aortic production of superoxide in arteries in abdominal aortic coarctation induced hypertensive rats and sham-operated normotensive rats, Scale bar = 100 μm. *n* = 3, Welch T test: *p = 0.0188, Hypertension group versus Sham group. **f-g** Superoxide production in rat VSMCs under static or cyclic stretch conditions was detected by DHE staining, Scale bar = 100 μm. *n* = 3, Welch T test: *p = 0.0364, Stretch group versus Static group. **h** Representative western blots of NOX1, NOX2 and NOX4 protein expression with β-actin serving as an internal control in aortas of SHRs and WKYs.** i** The quantification of NOX1 expression in aortas of SHRs and WKYs. *n* = 6, Welch T test: *p = 0.0268, SHR group versus WKY group. **j** The quantification of NOX2 expression in aortas of SHRs and WKYs. *n* = 6, Welch T test: *p* = 0.9290, SHR group versus WKY group.** k** The quantification of NOX4 expression in aortas of SHRs and WKYs. *n* = 6, Welch T test: *p = 0.0105, SHR group versus WKY group. **l** Representative western blots of NOX1, NOX2 and NOX4 protein expression with β-actin serving as an internal control in aortas of hypertensive and normotensive rats. **m** The quantification of NOX1 expression in aortas of hypertensive and normotensive rats. *n* = 3, Welch T test: *p = 0.0135, HTN group versus Sham group. **n** The quantification of NOX2 expression in aortas of abdominal aortic coarctation caused hypertensive rats and sham-operated normotensive rats. *n* = 3, Welch T test: *p = 0.0205, HTN group versus Sham group.** o** The quantification of NOX4 expression in aortas of abdominal aortic coarctation promoted hypertensive rats and sham-operated normotensive rats. *n* = 3, Welch T test: *p = 0.0188, HTN group versus Sham group. **p** CircLyst expression in rat VSMCs after H_2_O_2_ treatment. *n* = 3, Welch T test: **p = 0.0095, HTN group versus Sham group. **q** CircLyst expression was confirmed by qRT-PCR in VSMCs after transfection with siRNA-NOX1 under static or cyclic stretch conditions. The values are expressed by mean ± SD. *n* = 3, Welch T test: ***p = 0.0003, 15% stretch group versus Ctrl group, **p = 0.0064, transfection with siRNA-NOX1 under 15% stretch group versus 15% stretch group. **r** CircLyst expression was confirmed by qRT-PCR in VSMCs after transfection with siRNA-NOX4 under static or cyclic stretch conditions. The values are expressed by mean ± SD. *n* = 3, Welch T test: **p = 0.0001, 15% stretch group versus Ctrl group, **p = 0.0055, transfection with siRNA-NOX4 under 15% stretch group versus Ctrl group, **p = 0.0023, transfection with siRNA-NOX4 under 15% stretch group versus 15% stretch group

Reactive oxygen species (ROS) play important roles in mechanical stress induced mechanotransduction and vascular remodeling [24, 25, 42]. To explore the potential role of ROS in modulating non‑coding RNAs, we performed Ingenuity Pathway Analysis (IPA), a widely used tool for interpreting omics data in a biological context. IPA data indicated that there is crosstalk between reactive oxygen species (ROS) and non-coding RNAs (Fig. 2a). ROS regulate the expression of miRNAs such as mir-15, mir-21, mir-27, and mir-210, as well as circRNAs including circRNA-NFATC3 (Fig. 2a). Therefore, we investigated whether cyclic stretch induced ROS modulate circLyst expression. Dihydroethidium (DHE) staining was used to detect ROS production, and excessive ROS production was found in the aortas of SHRs compared to WKYs (Fig. 2b, c). Consistently, abdominal aorta coarctation induced hypertension also elevated aortic ROS production compared to sham control (Fig. 2d, e). Besides, cyclic stretch also induced rat VSMC ROS production in vitro (Fig. 2f, g).

Since NADPH oxidases (NOXs) are the main enzymatic sources of ROS during cardiovascular pathogenesis [4, 43], we detected NOX1, NOX2 and NOX4 expression in SHRs. NOX1 and NOX4, but not NOX2, were elevated in SHRs compared to WKYs (Fig. 2h-k). Similarly, NOX1 and NOX4 were increased while NOX2 was decreased in abdominal aorta coarctation induced hypertension (Fig. 2l-o). Of note, circLyst expression was significantly increased after H_2_O_2_ treatment for 24 h (Fig. 2p). To further explore the mediatory role of NOX1/4 in mechanical stretch-induced circLyst upregulation, VSMCs were transfected with specific siRNAs to knock down NOX1 and NOX4, respectively. Individual knockdown of NOX1 (Fig. 2q) and NOX4 (Fig. 2r) both significantly attenuated the stretch-induced upregulation of circLyst, confirming that NOX-ROS signaling is required for circLyst induction under high cyclic stretch.

Collectively, these data indicate that, in hypertension, high cyclic stretch activates NOX1 and NOX4 to generate ROS, thereby promoting circLyst expression.

### Silencing circLyst inhibits the proliferation and migration of vascular smooth muscle cells (VSMCs) and mesenteric smooth muscle cells (MSMCs)

**Fig. 3:**
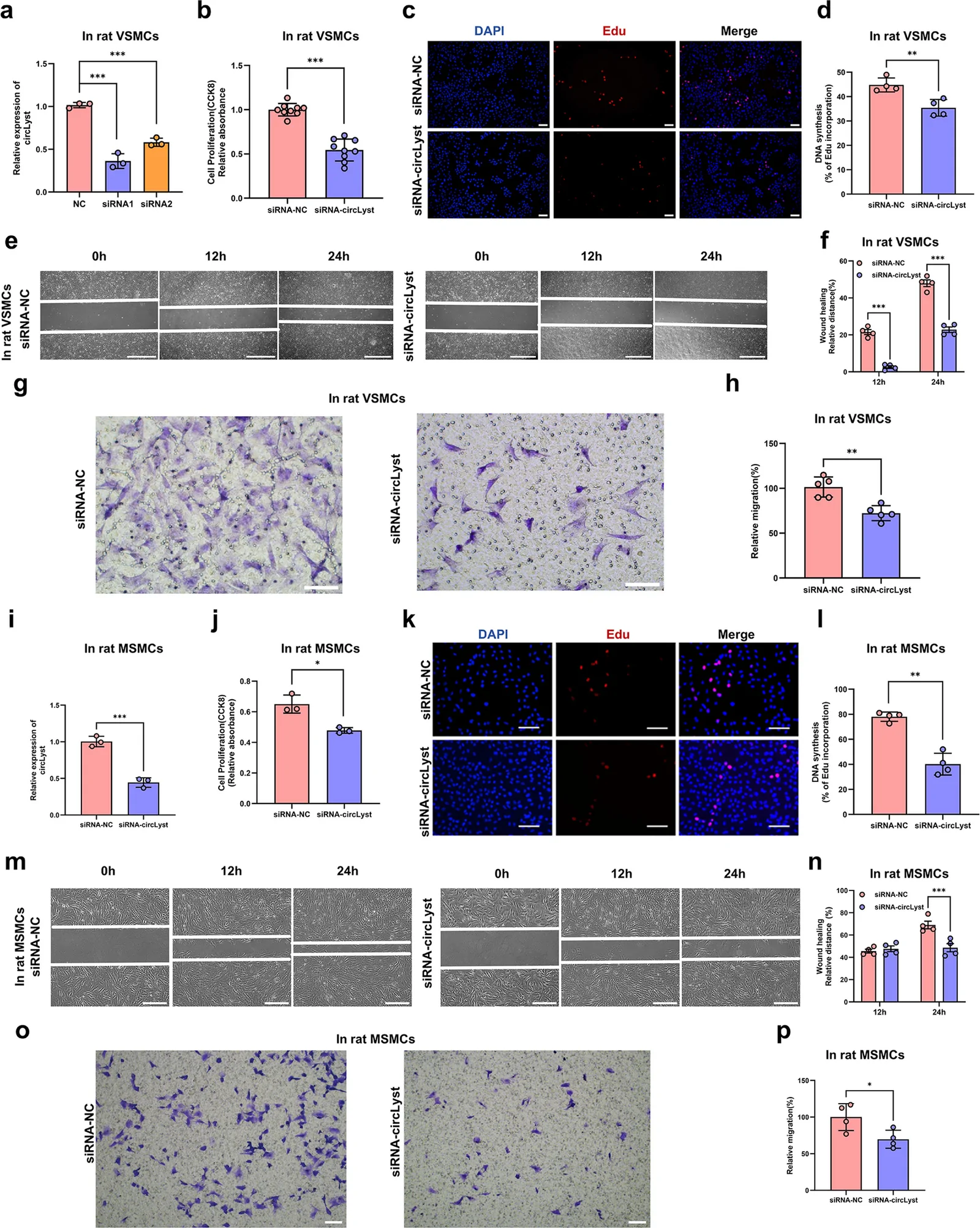
The silence of circLyst inhibited the proliferation and migration of VSMCs and MSMCs.** a** CircLyst expression was confirmed by qRT-PCR in VSMCs after transfection with circLyst-specific siRNA-1 or −2. The values are expressed by mean ± SD. *n* = 3, ANOVA followed by Tukey HSD test: ***p < 0.0001(siRNA1), ***p = 0.0003 (siRNA2), versus siRNA negative control (NC). **b** Knockdown of circLyst inhibited VSMC proliferation as detected by CCK-8 assay. The values are expressed by mean ± SD. *n* = 9, Welch T test: ***p < 0.0001, siRNA-circLyst group versus siRNA-NC group. **c** Knockdown of circLyst inhibited VSMC proliferation detected by EdU detection kit, Scale bar = 100 μm. **d** Quantitative EdU assay data from **c**. Data were presented as mean ± SD. *n* = 4, Welch T test: **p = 0.0062, siRNA-circLyst group versus siRNA-NC group. **e–f** Knockdown of circLyst inhibited VSMC migration detected by scratch healing assay, Scale bar = 50 μm. The results were expressed by mean ± SD. *n* = 4, Two-way ANOVA followed by Bonferroni-corrected Welch T tests: ***p < 0.001 (12 h), ***p < 0.001 (24 h), siRNA-circLyst group versus siRNA-NC group. **g-h** Knockdown of circLyst decreased VSMC migration determined by the modified Boyden chamber assay, Scale bar = 50 μm. The results were expressed by mean ± SD. *n* = 5, Welch T test: **p = 0.0020, siRNA-circLyst group versus siRNA-NC group.** i** CircLyst expression was confirmed by qRT-PCR in MSMCs after transfection with circLyst-specific siRNA-1. The values are expressed by mean ± SD. *n* = 3, Welch T test: ***p = 0.0006, siRNA-circLyst group versus siRNA-NC group. **j** Knockdown of circLyst significantly suppressed MSMC proliferation, as determined by CCK-8 assay. The values are expressed by mean ± SD. *n* = 3, Welch T test: *p = 0.0285, siRNA-circLyst group versus siRNA-NC group. **k** Knockdown of circLyst inhibited MSMC proliferation determined by EdU detection kit, Scale bar = 100 μm. **l** Quantitative EdU assay data from **k**. Data were presented as mean ± SD. *n* = 4, Welch T test: **p = 0.0013, siRNA-circLyst group versus siRNA-NC group. **m–n** Knockdown of circLyst inhibited MSMC migration detected by scratch healing assay, Scale bar = 50 μm. The results were expressed by mean ± SD. *n* = 4, Two-way ANOVA followed by Bonferroni-corrected Welch T tests: ***p = 0.0007 (24 h), siRNA-circLyst group versus siRNA-NC group. **o-p** Knockdown of circLyst decreased VSMC migration determined by the modified Boyden chamber assay, Scale bar = 100 μm. The results were expressed as mean ± SD. *n* = 4, Welch T test: *p = 0.0386, siRNA-circLyst group versus siRNA-NC group

To further evaluate the effects of circLyst on regulating VSMC function, circLyst-specific siRNAs were transfected into rat VSMCs (Fig. 3a). The siRNAs, especially siRNA1 robustly decreased the expression of circLyst. Then, we used siRNA1 to further validate the function of circLyst in VSMCs. The knockdown of circLyst significantly reduced the proliferation of VSMCs as evaluated by CCK-8 assay (Fig. 3b) and EdU assay (Fig. 3c, d). Meanwhile, we examined the effects of circLyst on cell migration using scratch wound healing assay and transwell assay. As shown in Fig. 3e, f, scratch wound healing assay showed that circLyst siRNA1 markedly decreased VSMC migration compared to siRNA-NC. Similarly, the transwell assay (Fig. 3g, h) also confirmed that VSMC migration was reduced by circLyst siRNA1 compared to siRNA-NC. Thus, circLyst ablation attenuated the proliferation and migration of VSMCs.

Besides, the roles of circLyst in MSMCs were also investigated. CircLyst-specific siRNA1 were successfully transfected into rat MSMCs (Fig. 3i). circLyst siRNAs significantly reduced the proliferation of MSMCs as detected by CCK-8 assay (Fig. 3j) and EdU assay (Fig. 3k, l). Furthermore, scratch wound healing assay and transwell assay were performed to explore the effect of circLyst on MSMC migration. As shown in Fig. 3m, n, scratch wound healing assay showed that circLyst siRNA1 markedly decreased MSMC migration compared to siRNA-NC. Similarly, the transwell assay (Fig. 3o, p) also confirmed that MSMC migration was reduced by circLyst siRNA1 compared to siRNA-NC.

These results imply that circLyst knockdown alleviated the proliferation and migration of both VSMCs and MSMCs.

### CircLyst regulates VSMC and MSMC proliferation and migration by sponging miR-298-5p

**Fig. 4:**
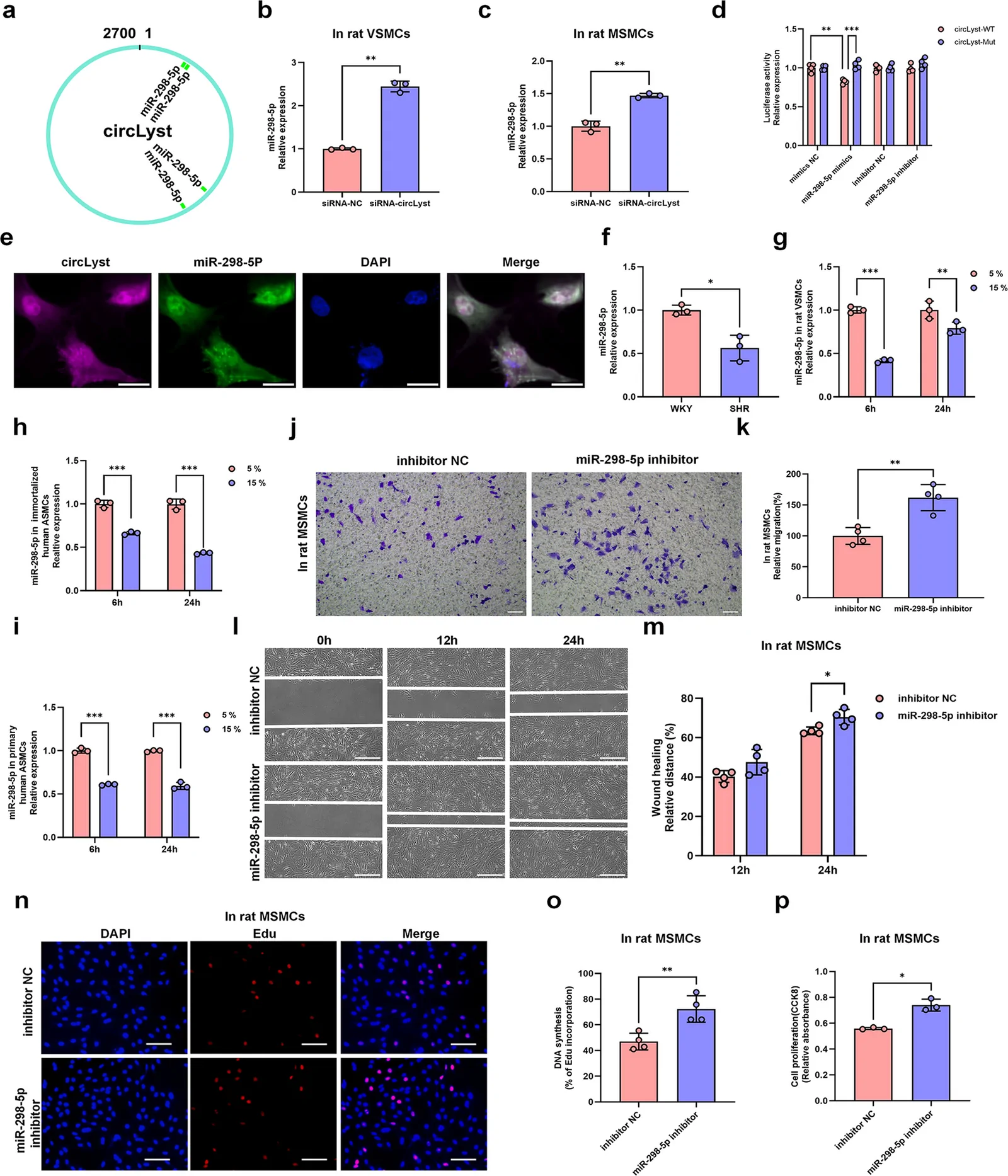
CircLyst sponges miR-298-5p to regulate SMC proliferation and migration. **a** miR-298-5p was predicted to bind to circLyst by miRranda website. **b** qRT-PCR verified the expression of miR-298-5p in VSMCs after transfection with circLyst-specific siRNA. The values are expressed by mean ± SD. *n* = 3, Welch T test: **p = 0.0019, siRNA-circLyst group versus siRNA-NC group. **c** qRT-PCR verified the expression of miR-298-5p in MSMCs after transfection with circLyst-specific siRNA. The values are expressed by mean ± SD. *n* = 3, Welch T test: **p = 0.0035, siRNA-circLyst group versus siRNA-NC group. **d** HEK-293 T cells were co-transfected with miR-298-5p mimic, mimics NC, miR-298-5p inhibitors or inhibitors NC and a luciferase reporter construct containing WT or (mutated) MUT circLyst. Data were presented as mean ± SD. *n* = 3, Two-way ANOVA followed by Bonferroni-corrected Welch T tests:**p = 0.001(circLyst-WT), miR-298-5p mimics group versus mimics NC group, ***P < 0.0001(miR-298-5p mimics), circLyst-Mut group versus circLyst-WT group. **e** FISH assay was used to detect the localization of circLyst and miR-298-5p in VSMCs, the images showing the co-localization of circLyst and miR-298-5p in VSMCs. miR-298-5p probes were labelled with FAM, whereas circLyst probes were tagged with Cy3. Nuclei were stained with DAPI. Scale bar = 20 µm.** f** The expression of miR-298-5p in the thoracic aorta of SHRs compared with WKYs detected by qRT-PCR. The values are expressed by mean ± SD, *n* = 3,Welch T test: *p = 0.0244, SHR group versus WKY group. **g** Rat VSMCs was harvested and subjected to 5% and 15% cyclic stretch respectively, miR-298-5p was detected in VSMCs under cyclic stretch for 6 h and 24 h respectively. *n* = 3, Two-way ANOVA followed by Bonferroni-corrected Welch T tests: ***p < 0.0001 (6 h), **p = 0.0082 (24 h), 15% cyclic stretch versus 5% cyclic stretch. **h** Immortalized human ASMCs were subjected to 5% and 15% cyclic stretch respectively, miR-298-5p was detected in ASMCs under cyclic stretch for 6 h and 24 h respectively. *n* = 3, Two-way ANOVA followed by Bonferroni-corrected Welch T tests: ***p < 0.0001 (6 h), ***p < 0.0001 (24 h), 15% cyclic stretch versus 5% cyclic stretch.** i** Primary human ASMCs were subjected to 5% and 15% cyclic stretch respectively, miR-298-5p was detected in ASMCs under cyclic stretch for 6 h and 24 h respectively. *n* = 3, Two-way ANOVA followed by Bonferroni-corrected Welch T tests: ***p < 0.0001 (6 h), ***p < 0.0001 (24 h), 15% cyclic stretch versus 5% cyclic stretch. **j-k** Knockdown of miR-298-5p increased MSMC migration determined by the modified Boyden chamber assay, Scale bar = 100 μm. The results were expressed by mean ± SD. *n* = 4, Welch T test: **p = 0.0041, miR-298-5p inhibitor group versus inhibitor NC group. **l-m** Knockdown of miR-298-5p induced MSMC migration detected by scratch healing assay, Scale bar = 100 μm. The results were expressed by mean ± SD. *n* = 4, Two-way ANOVA followed by Bonferroni-corrected Welch T tests: *p = 0.0363 (24 h), miR-298-5p inhibitor group versus inhibitor NC group. **n** Knockdown of miR-298-5p promoted MSMC proliferation detected by EdU detection kit Scale bar = 50 μm. **o** Quantitative EdU assay data from **n**. Data were presented as mean ± SD. *n* = 4, Welch T test: **p = 0.0084, miR-298-5p inhibitor group versus inhibitor NC group. **p** Knockdown of miR-298-5p significantly accelerated MSMC proliferation determined by CCK-8 assay. The values are expressed by mean ± SD. *n* = 3, Welch T test: *p = 0.0186, miR-298-5p inhibitor group versus inhibitor NC group

In cardiovascular system, acting as microRNA or protein inhibitors ('sponges') is an effective way for many circRNAs to exert their vital biological functions [34, 44, 45]. To explore if circLyst acts as a sponge for miRNAs, the miRNAs binding to circLyst were predicted using TargetScan, miRanda and RNAHybrid. It was found that miR-298-5p was the most likely potential target for circLyst since circLyst has four potential binding sites for miR-298-5p (Fig. 4a). miR-298-5p was upregulated both in VSMCs (Fig. 4b) and MSMCs (Fig. 4c) after transfection with circLyst siRNA1. Therefore, miR-298-5p could be a potential target of circLyst. To detect whether miR-298-5p directly binds to the circLyst, the circLyst sequence with wild type (WT) or mutant (Mut) miR-298-5p binding sites was inserted into pmirGLO vector (circLyst-WT or circLyst-Mut). Figure 4d showed that the relative luciferase activity of the reporter was reduced in the cells co-transfected with circLyst-WT and miR-298-5p mimics. However, the co-transfection of circLyst-WT and miR-298-5p inhibitors had no marked change in the relative luciferase activity. Moreover, circLyst-Mut has no significantly inhibitory effects on the luciferase activity. Therefore, circLyst is the competing endogenous RNA (ceRNA) of miR-298-5p to exhibits its biological function through regulation of miR-298-5p (Fig. 4d). Furthermore, RNA-FISH assay indicated that circLyst and miR-298-5p were co-localized in the cytoplasm of cells (Fig. 4e).

Notably, miR-298-5p was decreased in the thoracic aorta of SHR compared to that of WKY (Fig. 4f). miR-298-5p was also decreased significantly after 6 h and 24 h of 15% cyclic stretch compared to 5% cyclic stretch in rat VSMCs (Fig. 4g). The expression of miR-298-5p was also detected in immortalized human ASMCs. miR-298-5p was reduced significantly after 6 h and 24 h of 15% cyclic stretch (Fig. 4h). Besides, 15% cyclic stretch downregulated miR-298-5p expression both at the 6-h and 24-h time point in primary human ASMCs (Fig. 4i). Therefore, miR-298-5p was reduced by high cyclic stretch both in vitro and in vivo.

Next, we investigated the role of miR-298-5p in regulating MSMC migration and proliferation. miR-298-5p knockdown significantly enhanced MSMC migration, as confirmed by both the modified Boyden chamber assay (Fig. 4j, k) and scratch wound healing assay (Fig. 4l, m). Additionally, miR-298-5p depletion promoted MSMC proliferation, which was consistently observed using the EdU assay (Fig. 4n, o) and CCK-8 assay (Fig. 4p).

Collectively, our findings suggest that circLyst may function as a miR-298-5p sponge, thereby modulating SMC proliferation and migration in a miR-298-5p-mediated manner.

### Intermediate role of miR-298-5p in circLyst regulation of SMC proliferation and migration

**Fig. 5:**
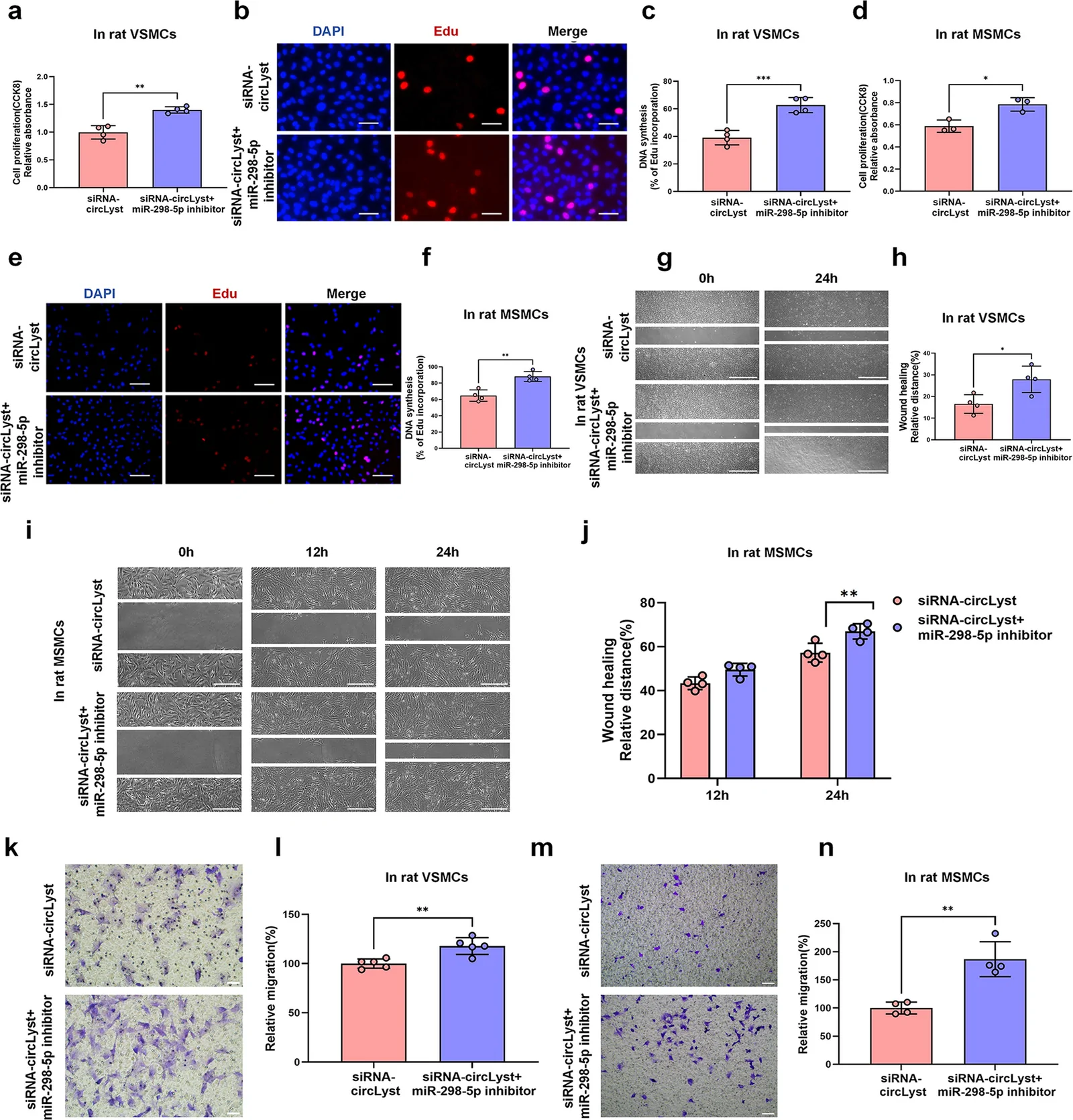
Blocking miR-298-5p restored SMC proliferation and migration suppressed by circLyst knockdown. **a** The transfection of miR-298-5p inhibitors restored VSMCs proliferation by CCK-8 assay. *n* = 4, Welch T test: **p = 0.0031, siRNA-circLyst + miR-298-5p inhibitor group versus siRNA-circLyst group.** b** The transfection of miR-298-5p inhibitors restored VSMCs proliferation detected by EdU detection kit, Scale bar = 50 μm. **c** Quantitative EdU assay data from **b**. Data were presented as mean ± SD. *n* = 4, Welch T test: ***P = 0.0008, siRNA-circLyst + miR-298-5p inhibitor group versus siRNA-circLyst group. **d** miR-298-5p inhibitors restored VSMCs proliferation detected by CCK-8 assay. *n* = 3, Welch T test: *p = 0.0147, siRNA-circLyst + miR-298-5p inhibitor group versus siRNA-circLyst group.** e** The transfection of miR-298-5p inhibitors restored VSMCs proliferation investigated by EdU detection kit, Scale bar = 100 μm. **f** Quantitative EdU assay data from **e**. Data were presented as mean ± SD. *n* = 4, Welch T test: **p = 0.0024, siRNA-circLyst + miR-298-5p inhibitor group versus siRNA-circLyst group. **g-h** The transfection of miR-298-5p inhibitors restored VSMCs migration detected by scratch healing assay, Scale bar = 50 μm. The results were expressed by mean ± SD. *n* = 4, Welch T test: *p = 0.0255, siRNA-circLyst + miR-298-5p inhibitor group versus siRNA-circLyst group. **i-j** miR-298-5p inhibitors restored MSMCs migration detected by scratch healing assay, Scale bar = 50 μm. The results were expressed by mean ± SD. *n* = 4, Two-way ANOVA followed by Bonferroni-corrected Welch T tests: **p = 0.0040 (24 h), siRNA-circLyst + miR-298-5p inhibitor group versus siRNA-circLyst group. **k-l** The transfection of miR-298-5p inhibitors restored VSMC migration determined by the modified Boyden chamber assay, Scale bar = 50 μm. The results were expressed by mean ± SD. *n* = 5, Welch T test: **p = 0.0059, siRNA-circLyst + miR-298-5p inhibitor group versus siRNA-circLyst group**. m–n** miR-298-5p inhibitors restored MSMC migration confirmed by the modified Boyden chamber assay, Scale bar = 100 μm. The results were expressed by mean ± SD. *n* = 4, Welch T test: **p = 0.0077, siRNA-circLyst + miR-298-5p inhibitor group versus siRNA-circLyst group

Since circLyst serves as a sponge for miR-298-5p, we verify the intermediate regulatory role of miR-298-5p in circLysts’ effect on cell functions. VSMCs were co-transfected with miR-298-5p inhibitors and circLyst-specific siRNAs. Compared to co-transfection of circLyst siRNA and inhibitor NC, the co-transfection of circLyst siRNA and miR-298-5p inhibitor increased VSMC proliferation detected by CCK-8 assay (Fig. 5a). Besides, EdU assay confirmed that miR-298-5p inhibitor also increased the proliferation of VSMC under circLyst siRNA transfection (Fig. 5b, c). In MSMCs, inhibition of miR-298-5p also significantly increased the proliferation rate, as demonstrated by both the CCK-8 assay (Fig. 5d) and EdU incorporation assay (Fig. 5e, f). These data suggested that miR-298-5p counteracts the effect of circLyst on SMC proliferation.

Furthermore, we also evaluated the intermediate regulatory role of miR-298-5p in circLyst’s effect on cell migration. Scratch wound healing assay (Fig. 5g, h) showed that miR-298-5p inhibitor induced the migration rate of VSMC under circLyst siRNA transfection. Similar results were obtained in MSMCs (Fig. 5i, j). Transwell assay was also performed to further access the role of miR-298-5p in VSMC migration, the data demonstrated that circLyst siRNA reduced VSMC migration rate was reversed by miR-298-5p inhibitor (Fig. 5k, l). Consistent with the above findings, a similar migration effect was observed in MSMCs upon miR-298-5p inhibition (Fig. 5m, n).

These results implied that circLyst regulates the proliferation and migration of SMCs via miR-298-5p.

### CircLyst-miR-298-5p axis modulates col23a1 and SMC phenotype under cyclic stretch

**Fig. 6:**
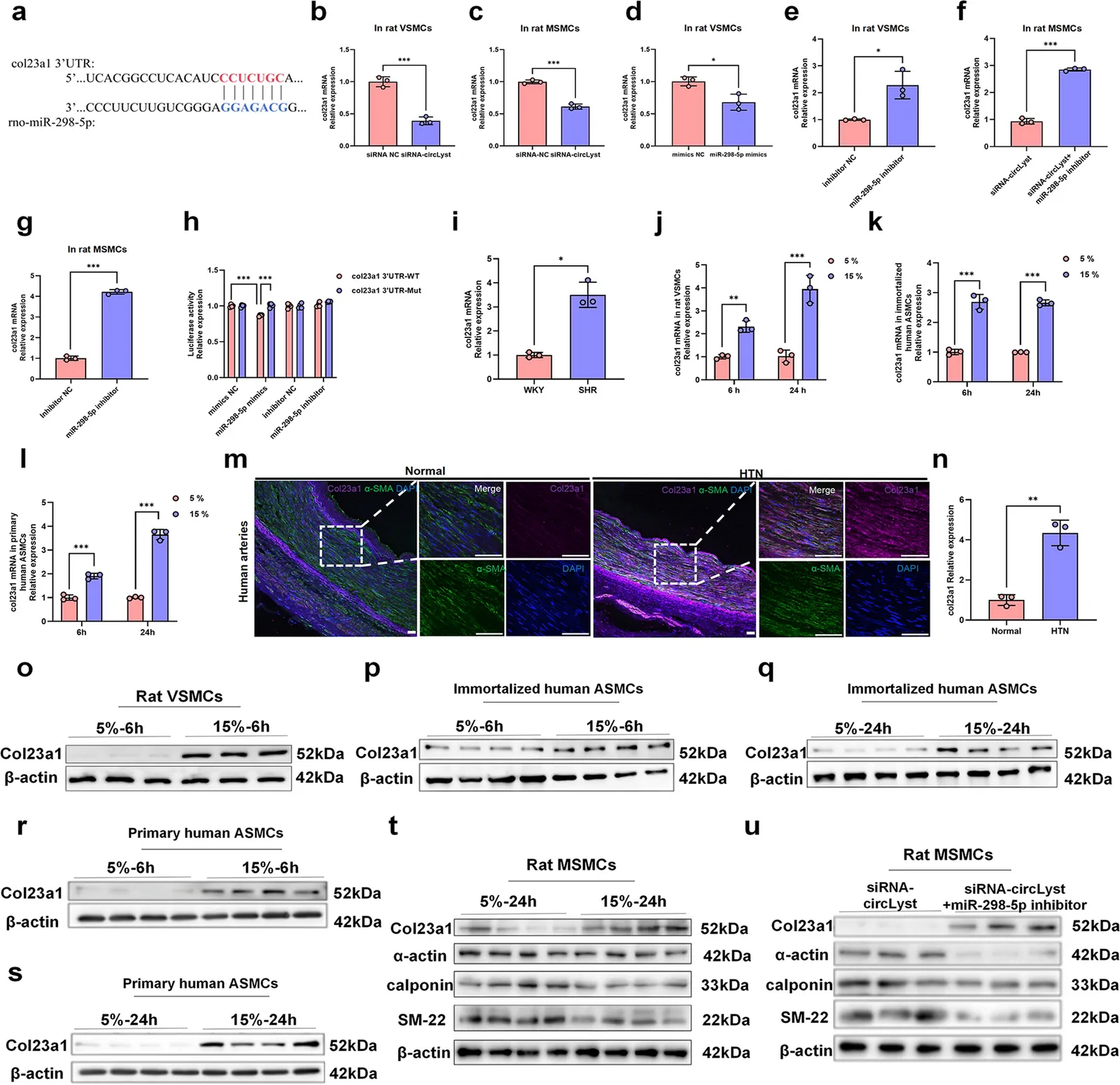
Col23a1 is regulated by miR-298-5p in SMCs and elevated under cyclic stretch in humans and rodents. **a** Schematic of the miR-298-5p putative binding site in col23a1 3′-UTR. **b** The expression of col23a1 was detected by qRT-PCR in VSMCs transfected with siRNA-circLyst and siRNA-NC. Data were presented as mean ± SD. *n* = 3, Welch T test: ***p = 0.0007, siRNA-circLyst group versus siRNA NC group. **c** The expression of col23a1 was detected by qRT-PCR in MSMCs after siRNA-circLyst transfection. Data were presented as mean ± SD. *n* = 3, Welch T test: ***p = 0.0003, siRNA-circLyst group versus siRNA NC group. **d-e** The expression of col23a1 was detected by qRT-PCR in VSMCs transfected with miR-298-5p mimics or inhibitors. Data were presented as mean ± SD. *n* = 3, Welch T test: *p = 0.0271, miR-298-5p mimics group versus mimics NC group; *n* = 3, Welch T test: *p = 0.0485, miR-298-5p inhibitor group versus inhibitor NC group.** f** The expression of col23a1 in MSMCs after miR-298-5p inhibitors and siRNA-circLyst co-transfection. Data were presented as mean ± SD. *n* = 3, Welch T test: ***p < 0.0001, siRNA-circLyst + miR-298-5p inhibitor group versus siRNA-circLyst group.** g** The expression of col23a1 in MSMCs after miR-298-5p inhibitors transfection. Data were presented as mean ± SD. *n* = 3, Welch T test: ***P < 0.0001, miR-298-5p inhibitor group versus inhibitor NC group.** h** HEK-293 T cells were co-transfected with miR-298-5p mimics, mimics NC, miR-298-5p inhibitors or inhibitors NC and a luciferase reporter construct containing WT or MUT col23a1. Data were presented as mean ± SD. *n* = 3, Two-way ANOVA followed by Bonferroni-corrected Welch T tests: ***p < 0.0001 (col23a1 3'UTR-WT), miR-298-5p mimics group versus mimics NC group, ***p < 0.0001 (miR-298-5p mimics), col23a1 3'UTR-Mut group versus col23a1 3'UTR-WT group**. i** The expression of col23a1 was detected by qRT-PCR in the thoracic aortas of SHRs and WKYs. Data were presented as mean ± SD. *n* = 3, Welch T test: *p = 0.0119, SHR group WKY group**. j** Rat VSMCs was harvested and subjected to 5% or 15% cyclic stretch respectively, col23a1 was detected in VSMCs under cyclic stretch for 6 h and 24 h respectively. *n* = 3, Two-way ANOVA followed by Bonferroni-corrected Welch T tests: **p = 0.0038 (6 h), ***p < 0.0001 (24 h), 15% cyclic stretch versus 5% cyclic stretch**. k** Immortalized human ASMCs were subjected to 5% or 15% cyclic stretch respectively, col23a1 was detected in ASMCs under cyclic stretch for 6 h and 24 h respectively. *n* = 3, Two-way ANOVA followed by Bonferroni-corrected Welch T tests: ***p < 0.0001 (6 h), ***p < 0.0001 (24 h), 15% cyclic stretch versus 5% cyclic stretch**. l** Primary human ASMCs were subjected to 5% or 15% cyclic stretch respectively, col23a1 was detected in ASMCs under cyclic stretch for 6 h and 24 h respectively. *n* = 3, Two-way ANOVA followed by Bonferroni-corrected Welch T tests: ***p < 0.0001 (6 h), ***p < 0.0001 (24 h), 15% cyclic stretch versus 5% cyclic stretch. **m–n** Representative immunofluorescence of col23a1 and α-SMA in arteries of hypertensive and normotensive human, Scale bar = 100 μm. *n* = 3, Welch T test: **P = 0.0052, HTN group versus Normal group. **o** Rat VSMCs was harvested and subjected to 5% or 15% cyclic stretch respectively, col23a1 protein expression was detected in VSMCs under cyclic stretch for 6 h. **p** Immortalized human ASMCs were subjected to 5% or 15% cyclic stretch respectively, col23a1 protein expression was detected in ASMCs under cyclic stretch for 6 h. **q** Immortalized human ASMCs were subjected to 5% or 15% cyclic stretch respectively, col23a1 protein expression was detected in ASMCs under cyclic stretch for 24 h. **r** Primary human ASMCs were subjected to 5% or 15% cyclic stretch respectively, col23a1 protein expression was detected in ASMCs under cyclic stretch for 6 h. **s** Primary human ASMCs were subjected to 5% or 15% cyclic stretch respectively, col23a1 protein expression was detected in ASMCs under cyclic stretch for 24 h. **t** Rat MSMCs was subjected to 5% or 15% cyclic stretch respectively. Col23a1, α-actin, calponin and SM-22 were detected in MSMCs under cyclic stretch for 24 h. **u** Rat MSMCs was co-transfected with miR-298-5p inhibitors and siRNA-circLyst. Col23a1, α-actin, calponin and SM-22 were detected in MSMCs after co-transfection.

To explore the downstream molecule of miR-298-5p and the role of circLyst-miR-298-5p axis in regulating SMC phenotype, bioinformatics analysis was performed. Collagen type XXIII alpha 1(col23a1) was predicted to be a putative target of miR-298-5p (Fig. 6a) by the algorithms using miRanda and TargetScan. The silence of circLyst reduced the level of col23a1 both in VSMCs (Fig. 6b) and MSMCs (Fig. 6c).

Besides, miR-298-5p mimics decreased the level of col23a1 (Fig. 6d) while miR-298-5p inhibitors increased col23a1 expression (Fig. 6e) in VSMCs. Consistently, miR-298-5p overexpression diminished col23a1 expression (Fig. 6f), while its antagonism significantly upregulated col23a1 expression (Fig. 6g) in MSMCs. The dual luciferase reporter assay was used to further validate the potential relationship between miR-298-5p and col23a1. miR-298-5p mimics significantly inhibited the luciferase activity in cells transfected with col23a1-WT-Report, but not in those transfected with col23a1-MUT-Report (Fig. 6h). Transfection of VSMCs with miR-298-5p mimics at concentrations of 50, 100, and 200 µM resulted in a significant increase in miR-298-5p levels (Fig. S1a) and a marked reduction in circLyst expression (Fig. S1b) across all doses. Furthermore, col23a1 expression (Fig. S1c) was significantly downregulated by the 100 and 200 µM mimic concentrations. These data indicated that col23a1 is the direct target of miR-298-5p.

We next examined the expression of col23a1 in the aortas of hypertensive rats and VSMCs under high cyclic stretch. PCR results showed that col23a1 was increased in the thoracic aortas of SHRs compared to those of WKYs (Fig. 6i). Compared with 5% cyclic stretch, 15% cyclic stretch increased the mRNA level of col23a1 in rat VSMCs both at 6 h and 24 h time points (Fig. 6j). Besides, the expression of col23a1 mRNA was detected in immortalized human ASMCs and primary human ASMCs under high cyclic stretch. Col23a1 mRNA expression was significantly upregulated following 6 h and 24 h of 15% cyclic stretch in both immortalized (Fig. 6k) and primary (Fig. 6l) human ASMCs.

Of note, col23a1 protein expression was also examined in hypertensive human arteries. Immunofluorescence staining indicated that col23a1 protein was increased in hypertensive human arteries compared to normotensive arteries (Fig. 6m, n). Besides, 15% cyclic stretch also increased col23a1 protein expression in rat VSMCs after 6 h stimulation compared to 5% cyclic stretch (Fig. 6o, Fig. S2a). Col23a1 protein expression was also elevated in immortalized human ASMCs after 6 h (Fig. 6p, Fig. S2b) and 24 h (Fig. 6q, Fig. S2c) of 15% cyclic stretch compared to 5% cyclic stretch. Furthermore, the protein expression of col23a1 was also detected in primary human ASMCs subjected to high cyclic stretch. Col23a1 protein was induced by 15% cyclic stretch after 6 h (Fig. 6r, Fig. S2d) and 24 h (Fig. 6s, Fig. S2e) application. Notably, 15% cyclic stretch significantly upregulated Col23a1 protein expression in rat MSMCs after 24 h stimulation compared to 5% stretch, while concurrently downregulated SMC differentiation markers (α-actin, calponin, and SM-22α) (Fig. 6t, Fig. S2f). Besides, the co-transfection of circLyst siRNA and miR-298-5p inhibitor also induced col23a1 protein expression while reduced α-actin, Calponin and SM-22α expression compared to co-transfection of circLyst siRNA and inhibitor NC in MSMCs (Fig. 6u, Fig. S2g).

Therefore, circLyst-miR-298-5p axis induces col23a1 expression and promotes SMC dedifferentiation (synthetic VSMCs, with a high migration and proliferation capacity) under high cyclic stretch.

### CircLyst ablation reduces vascular remodeling

**Fig. 7:**
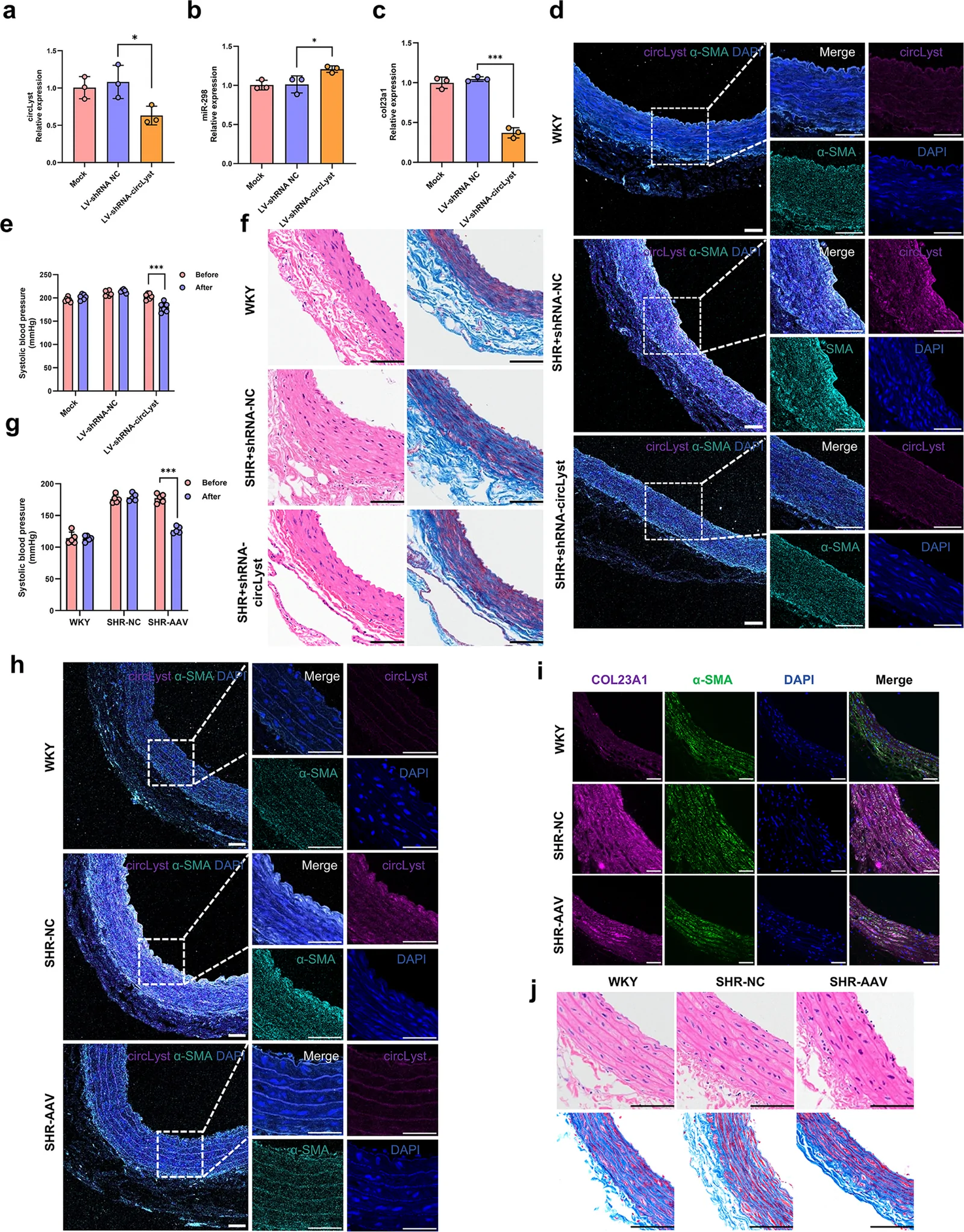
The silence of circLyst attenuated vascular remodeling and hypertension.** a** The expression of circLyst was detected by qRT-PCR in the SD rat thoracic aortas transfected with Lentivirus (LV)-shRNA-circLyst or LV-shRNA-NC. Data were presented as mean ± SD. *n* = 3, ANOVA followed by Tukey HSD test: *p = 0.0412, LV-shRNA-circLyst versus LV-shRNA NC. **b** The expression of miR-298-5p was detected by qRT-PCR in the SD rat thoracic aortas transfected with LV-shRNA-circLyst or LV-shRNA-NC. Data were presented as mean ± SD. *n* = 3, ANOVA followed by Tukey HSD test: *p = 0.0451, LV-shRNA-circLyst versus LV-shRNA NC.** c** The expression of col23a1 was detected by qRT-PCR in thoracic aortas of SD rats after transfection with LV-shRNA-circLyst or LV-shRNA-NC. Data were presented as mean ± SD. *n* = 3, ANOVA followed by Tukey HSD test: ***p < 0.0001, LV-shRNA-circLyst versus LV-shRNA NC. **d** FISH assay was performed to detect circLyst (Cy3-labeled probes) and α-SMA (FAM-labeled probes) expression in aortas of SHRs and WKYs. Nuclei were stained with DAPI, Scale bar = 50 μm. **e** Systolic blood pressure was measured after transfection with LV-shRNA-circLyst or LV-shRNA-NC. *n* = 6, two-way ANOVA followed by Bonferroni-corrected Welch T tests. ***p < 0.0001. **f** Representative Hematoxylin and eosin staining (H&E staining) and Masson's trichrome staining images of abdominal aortas from WKY, SHR + NC and SHR + LV-shRNA-circLyst groups, Scale bar = 100 μm.** g** Systolic blood pressure was measured after transfection with AAV-RNAi-circLyst or AAV-RNAi-NC. *n* = 5, two-way ANOVA followed by Bonferroni-corrected Welch T tests: ***p < 0.0001(SHR-AAV), before versus after transfection with AAV. **h** FISH assay was performed to detect circLyst (Cy3-labeled probes) and α-SMA (FAM-labeled probes) expression in abdominal aortas of SHRs and WKYs. Nuclei were stained with DAPI, Scale bar = 50 μm. **i** Representative immunofluorescence staining of col23a1 and α-SMA in abdominal aortas of WKY, SHR + NC and SHR + AAV-RNAi-circLyst groups, Scale bar = 50 μm.** j** Representative H&E and Masson's trichrome staining images of abdominal aortas from WKY, SHR + NC and SHR + AAV-RNAi-circLyst groups, Scale bar = 100 μm

To explore the role of circLyst in vascular remodeling in vivo, the recombinant lentivirus vector-expressing circLyst shRNA (LV-shRNA-circLyst) or negative control shRNA viruses (LV-shRNA-NC) were constructed. Then, LV-shRNA-circLyst or LV-shRNA-NC were delivered to the arteries of the SHRs as previously described [46]. CircLyst was effectively inhibited in arteries by LV-shRNA-circLyst compared to LV-shRNA-NC four weeks after transfection (Fig. 7a). PCR data showed that LV-shRNA-circLyst significantly increased the expression of miR-298-5p (Fig. 7b) and inhibited the expression of col23a1 in vivo (Fig. 7c). FISH assay confirmed that LV-shRNA-circLyst effectively reduced the elevated circLyst expression in the abdominal aortas of SHR (Fig. 7d). Besides, systolic blood pressure was attenuated in SHRs after transfection of LV-shRNA-circLyst compared to LV-shRNA-NC (Fig. 7e). Notably, hypertension induced vascular remodeling was also abolished by LV-shRNA-circLyst (Fig. 7f).

Furthermore, VSMC-specific knockdown of circLyst via AAV (AAV-RNAi-circLyst) was conducted to validate its functional role in vivo. AAV-RNAi-circLyst significantly lowered blood pressure in SHR (Fig. 7g). FISH assays demonstrated the efficacy of AAV-RNAi-circLyst in silencing circLyst in abdominal aortas of SHR (Fig. 7h). AAV-RNAi-circLyst treatment also significantly decreased the upregulated Col23a1 protein levels in abdominal aortas (Fig. 7i). Moreover, H&E and Masson’s trichrome staining revealed that LV-shRNA-circLyst effectively prevented vascular remodeling induced by hypertension (Fig. 7j).

Together, these findings imply that circLyst, which is upregulated by high cyclic stress‑induced oxidative stress, may contribute to vascular remodeling in hypertensive humans and rodents.

## Discussion

In the current study, we have identified a novel circRNA, termed circ_2465 (circLyst), and suggest for the first time that circLyst may serve as a regulator of vascular remodeling in humans and rodents. High cyclic stretch-induced ROS appear to promote the circLyst/miR-298-5p axis in vascular remodeling during hypertension. Additionally, circLyst was also found to be elevated in the plasma of hypertensive patients, and high cyclic stretch-induced ROS may be an important contributor in this process. Notably, inhibition of circLyst, either globally or specifically in VSMCs, shows potential therapeutic efficacy by attenuating vascular remodeling and improving hypertensive phenotypes. Taken together, our findings point to a potential role for the circLyst/miR-298-5p axis in oxidative stress-induced vascular remodeling under cyclic stretch. Targeting circLyst could represent a candidate strategy for hypertension management. However, the present study provides initial evidence that plasma circLyst is elevated in hypertensive patients based on a pilot cohort of 28 subjects (17 controls and 11 patients). Therefore, independent validation in a larger, multicenter, prospective cohort is warranted before considering clinical application of circLyst.

MicroRNAs (miRNAs), long noncoding RNAs (lncRNAs), and circular RNAs (circRNAs) are the three mains noncoding RNAs (ncRNAs) that emerging as relevant contributors to cardiovascular diseases [47]. Others and we have shown that mechanical stress is an important stimulus in vascular remodeling in hypertension [28, 48–51], miRNAs and lncRNAs were involved in this process [5, 28, 48, 52, 53]. We have investigated the roles of abnormal cyclic stretch and vascular injury in vascular remodeling and dysfunction [41, 54, 55] and proved that high cyclic stretch modulated miRNAs including miR-124-3p [5], miR-27a [28] and miR-142–3p [48] are robust regulators of vascular remodeling in hypertension. LncRNA XR007793 was found to regulate cyclic stretch-induced proliferation and migration of VSMCs in hypertension [52]. However, the role and mechanism of mechanical stress regulated circRNA in hypertension has not been reported yet. Here, we have identified a mechanosensitive circRNA, circLyst, in both hypertensive rats and humans. Our data suggest that targeting circLyst might be effective in attenuating vascular remodeling and hypertension in rodent models. Hence, inhibition of circLyst could potentially serve as a candidate strategy for managing hypertension in humans, though further validation is required.

Several studies have shown that circRNAs play important roles in vascular diseases [45, 56]. Circ_Lrp6, a circRNA enriched in VSMCs, acts as a sponge regulating miRNA-145 function [56]. The differential expression of miR-145 and circ_Lrp6 in murine and human vascular diseases implied that the imbalance between the bound and unbound forms of circ_Lrp6 to miR-145 could potentially contribute to vascular pathogenesis [56]. Zeng et al. discovered that circular RNA circMAP3K5 acts as a microRNA-22-3p sponge, promoting the resolution of intimal hyperplasia through TET2-mediated smooth muscle cell differentiation [45]. CircRNA Galntl6 was found to be a novel factor that attenuate stress-induced hypertension, and the circRNA Galntl6/miR-335/Lig3 axis played a vital role in this process [57]. CircEsyt2 was proved to directly interact with polyC-binding protein 1 to regulate VSMCs via splicing regulation in atherosclerotic mouse models [58]. Besides, circKrt4 [59], circSMOC1 [60] and Circ-Calm4 [61] regulate pulmonary vascular remodeling in pulmonary arterial hypertension while lower circGSAP [62] level is associated with the development of idiopathic pulmonary arterial hypertension and worse outcomes. However, the role of cyclic stretch in modulating circRNAs in hypertension was not clarified yet. Here, we have identified a novel circRNA, circLyst, that appears to respond to cyclic stretch in both rat and human VSMCs in vitro. In addition, cyclic stretch-induced oxidative stress may contribute to the upregulation of circLyst, which might subsequently activate miR-298-5p in hypertensive subjects. Of note, circLyst appears to have a relatively high stability compared with mRNA, and its modulation might be more feasible than directly manipulating cyclic stretch or oxidative stress. Taken together, circLyst may be considered a candidate target for further investigation in vascular remodeling and hypertension.

Excessive ROS have been proved to be an important mediator in endothelial dysfunction and vascular remodeling [43, 63–66]. Mounting evidence demonstrates that aberrant shear stress and heightened cyclic stretch trigger ROS accumulation via activating different ROS enzymatic sources. In the context of ventilator-induced lung injury, high cyclic stretch triggers a NOX2-specific (vs. NOX1/NOX4) response, culminating in lung injury via downstream activation of the ROS/CaMKII/ERK1/2 signaling axis [23, 64]. In cell-based models of atherosclerosis, exposure to cyclic stretch (15% strain at 0.05 Hz) upregulated both NOX1 expression and ROS production in VSMCs [67]. In turn, oxidative stress produced by NOX can also impair exercise capacity. Endothelial NOX1, but not NOX2 or NOX4, triggers mitochondrial dysfunction in skeletal muscle, compromising both exercise performance and energy metabolism, and consequently fostering the development of obesity and metabolic syndrome [68]. Here, we demonstrate that NOX1 and NOX4—but not NOX2—are upregulated in spontaneously hypertensive rats (SHR) and in abdominal aorta coarctation–induced hypertension, relative to their respective normotensive controls. CircLyst was also elevated by cyclic stretch-induced ROS in this process. DHE staining was used to semi-quantitative measure of overall ROS production in this study. However, this method lacks the specificity to differentiate between O₂⁻ and H₂O₂, and it also fails to quantitatively establish the dose–response relationship with circLyst expression. The quantitative link between ROS levels and circLyst expression warrants further investigation.

In conclusion, our findings point to a novel circRNA, circLyst, that appears to participate in the regulation of VSMC proliferation and migration, possibly through miR-298-5p mediated by oxidative stress under cyclic stretch. These observations might provide additional insights into the molecular basis of mechanotransduction in hypertensive vascular remodeling. Of note, circLyst could be considered a candidate for further exploration in the context of preventing vascular remodeling and hypertension.

## Materials and methods

### Rat models of hypertension and in vivo blood pressure monitoring

The spontaneously hypertensive rats (SHRs) and normotensive Wistar–Kyoto rats (WKYs) (12 weeks old, 250–300 g) were purchased from Beijing Vital River Laboratory Animal Technology Company (Beijing, China), and Sprague Dawley (SD) rats were purchased from Shanghai Laboratory Animal Research Center (Shanghai, China). Hypertensive rat model with abdominal aortic coarctation was generated as previously described [25]. Briefly, male SD rats (200 ± 20 g) were randomly assigned to sham-operated or abdominal aorta-constriction group. After anesthesia with isoflurane inhalation, the abdominal cavity was carefully incised to facilitate the separation of the abdominal aorta. At approximately 5 mm superior to the bifurcation of the right renal artery, the aorta was tied with a 21-gauge needle using a 3–0 surgical suture, then the needle was retrieved to create the aortic constriction with a diameter of about 0.9 mm. For sham-operated groups, the same procedure was operated but without constriction. Blood pressure was measured two weeks later in both groups by inserting a catheter into the common carotid arteries. Systolic blood pressure of SHRs and WKYs was recorded prior to the experiment and once a week using tail-cuff plethysmography via the BP-2010A apparatus (Softron Biotechnology Ltd, Beijing, China). The Blood pressure was averaged from six successive measurements.

All animals were housed in a temperature-controlled room with a 12-h light/dark cycle and free access to standard chow and water. All experimental protocols were approved by the Institutional Animal Care and Use Committee of Shanghai Jiao Tong University (Shanghai, China) and the Ethics Committees of Shanghai Jiao Tong University, and all experiments were performed in accordance with the recommendations of the Animal Management Rules of China (Documentation 55, 2001, Ministry of Health, China).

### The collection of human blood samples and aortic samples

Human blood samples were collected to detect the expression of circLyst, human aortic samples were harvested to detect Col23a1 expression.

This study included blood samples from hypertensive patients at the Third Hospital of Changsha, Changsha City, Hunan Province, China, and the Second Affiliated Hospital of Shaanxi University of Chinese Medicine, Xianyang City, Shaanxi Province, China, collected from July 2023 to March 2024 (Table S2). The study subjects were patients diagnosed with primary hypertension, characterized by a blood pressure of 140/90 mmHg or higher. No patients with secondary hypertension (i.e., caused by renal, endocrine, vascular, or cerebrovascular diseases) were included in this study. The control group consisted of healthy volunteers with normal blood pressure from Xianyang, Shaanxi, and Changsha, Hunan, China. Peripheral venous blood samples were collected after an overnight fast of 12 h and were immediately placed into an RNA preservation reagent (Genestonebio, Jiangsu, China). Differential hsa-circLyst expression was analyzed in the peripheral blood samples from hypertensive patients and the control group by qRT-PCR as mentioned above. This study was approved by the Ethics Committee of the Third Hospital of Changsha, Changsha, Hunan Province, China (no. KY-EC-2023–009) and the Second Affiliated Hospital of Shaanxi University of Traditional Chinese Medicine, Xianyang, Shaanxi Province, China (no. SZEFYIEC-KYPJ-2024001–2).

Human hypertensive and normotensive arterial samples were collected from patients with nephroblastoma. These patients underwent kidney tumor removal surgery at Shanghai Xinhua Hospital, and renal artery specimens were preserved thereafter. The project involving human hypertensive artery sample and normotensive control sample was approved by the Ethics Committee of Shanghai Xinhua Hospital, Shanghai Jiao Tong University, School of Medicine (approval ID: XHEC-D-2024–002).

### Detection of circRNA by next-generation sequencing and quantitative real-time PCR (qRT-PCR)

Next-generation sequencing was performed at an Illumina Hiseq™ 2500 platform, Oebiotech Institute (China). CIRI software was used to predict circRNAs [70]. Differentially expressed genes, including circRNAs and mRNAs, in the SHRs and WKYs, were analyzed utilizing DESeq with the following criteria: fold change > 2 and *p*-value (*p*) < 0.05. The Miranda package was used to predict the miRNA target at the 3′UTR region of differentially expressed mRNAs and the full-length sequence of differentially expressed circRNAs [71]. Competing endogenous RNA (ceRNA) relationships were generated by a circRNA and mRNA pair affected by the same miRNA members.

The stability and half-life of circRNAs were tested by RNase R and actinomycin D treatment assays. Total RNA (1 μg) was incubated with RNase R (2 U; Epicenter, Madison, WI, USA) at 37 °C for 10 min, and the reaction was stopped at 70 °C for 10 min. Then, circRNA and the corresponding linear mRNA were detected by qRT-PCR (PCR primer sequences of circRNAs and mRNA were shown in Table S3). 2 μmol/mL Actinomycin D (MilliporeSigma, Burlington, MA, USA) or DMSO was added to the medium of VSMCs. At different intervals, circRNA and the corresponding linear mRNA were detected by qRT-PCR.

### Cyclic stretch application

Cyclic stretch was performed as we previously described [5, 16, 22, 28, 41, 69]. Cells were plated on gelatin-coated flexible silicone bottom plates (Flexcell International, USA) at a density of 3 × 10^5^ cells per well (9.32 cm^2^). Twenty-four hours after seeding, VSMCs were incubated with DMEM for 24 h to arrest growth and synchronize the cells. As shown in Fig. 1K, the cells were subjected to cyclic stretch, which was produced by a computer-controlled vacuum (FX-5000 T Strain Unit, Flexcell International, USA). The following mechanical parameters were applied: (i) a stretch magnitude of 5% to mimic normal arterial mechanical condition or 15% to mimic hypertensive mechanical condition, (ii) a frequency of 1.25 Hz, and (iii) a duration of 6 h or 24 h.

### Ingenuity Pathway Analysis (IPA)

The crosstalk between reactive oxygen species (ROS) and non-coding RNAs including circRNAs and miRNAs were analyzed by QIAGEN Ingenuity Pathway Analysis (QIAGEN IPA, content version: 111,725,566, https://digitalinsights.qiagen.com/products-overview/discovery-insights-portfolio/analysis-and-visualization/qiagen-ipa/).

### Cell transfection

The siRNAs targeting the back-splicing junction of circLyst were designed and synthesized by Gene Pharma (Shanghai, China), the sequences of circLyst siRNAs and siRNA negative control (NC) were shown in Table S4. The mimics and inhibitors of miR-298-5p were synthesized by RiboBio (Guangzhou, China), the sequences of miR-298-5p mimics and inhibitors and their respective NC were shown in Table S5. Oligonucleotides were transfected into VSMCs respectively with Lipofectamine 3000 following the manufacturer's protocol. After 48 h, cells were harvested and lysed for qRT-PCR or western blot analysis.

### Knockdown circLyst in SHR

The recombinant RNAi plasmids were constructed by GenePharma (China). The lentiviral particles LV-shRNA-circLyst and LV-shRNA-NC were packaged using GM easy™ lentiviral Packaging Kit (Genomeditech, China). An established method was used for local delivery of the shRNA of circLyst lentivirus (LV-shRNA-circLyst) and negative control (LV-shRNA-NC) into aortas with Pluronic F-127 gel (Sigma, USA) as previously described. Rats were randomly divided into three groups including the LV-shRNA-circLyst-treated group, the LV-shRNA-NC-treated group, and the mock group. The sequences of circ_2465 shRNA and shNC were shown in Table S6. The aortas were collected 4 weeks after surgery.

RNAi-custom-adeno-associated virus called AAV9-circ_2465 (circLyst)-RNAi (P24M0086) was constructed by Shanghai Genechem Co., Ltd for specific knockdown of VSMC circLyst (AAV-RNAi-circLyst). Following the package, AAV-9 was administrated via intravenous injection on day 1 (30 μL, 4.45E + 12 v.g. mL^− 1^). Three weeks later, the rats were sacrificed and the vessels were harvested for further detection.

### RNA fluorescence in situ hybridization (FISH)

Fluorescence in situ hybridization (FISH) was employed to confirm the cytoplasmic localization of circLyst in VSMCs, to verify its elevated expression in hypertensive models, to demonstrate its co-localization with miR-298-5p, and to validate the knockdown efficacy of both LV-shRNA-circLyst and AAV-RNAi-circLyst in vivo.

The specific probes targeting circLyst, miR-298-5p, or α-actin were designed and synthesized by Shanghai GenePharma Biotechnology Co., Ltd. Following the instructions of the RNA FISH kit, their expression in both cells and paraffin-embedded tissue sections was detected using the "SA-Biotin Amplification System for Cell Climbing Slides" and "SA-Biotin Amplification System for Paraffin Sections" kits (GenePharma, Shanghai, China), respectively. Briefly, cells were seeded into 12-well confocal dish, while paraffin sections were processed with eco-friendly dewaxing solution prior to experimentation. For double FISH assay, cell slides or paraffin-embedded tissue sections were hybridized with circLyst probe (cy3-labelled) and miR-298-5p probe (FAM-labelled), or circLyst probe (cy3-labelled) and α-actin probe (FAM-labelled) in a ratio of 8 μl probe and 2 μl SA at 37 °C overnight. Nuclei were stained with 4,6-diamidino-2-phenylindole (DAPI). The probe sequence complementary to the junction part of circLyst was 5’-AACCTTCAAGAAGGGTTTGTGGGGA-3’, and the probe sequence complementary to miR-298-5p was 5’-GGGAAGAACAGCCCTCCTCTGCC-3’. α-SMA-rat probe was 5'-AAAACAGCCCTGGGAGCATCATC-3', 5'-GTCCAGCACAATACCAGTTGTACGT-3', and 5'-CAGCAGTAG + TCACGAAGGAATAGCC-3'. The amplification of the signal was based on the SA-Cy3 or FAM system. All procedures were conducted in a dark environment to prevent photobleaching. Images were captured by Leica Steliaris 5 (Leica, Germany).

### Dual-luciferase reporter assay

Dual-luciferase reporter assays were carried out to assess the binding between circLyst and miR-298-5p, and between miR-298-5p and col23a1, respectively. The Wild-type (WT) circLyst or the 3′ untranslated region (3′ UTR) of col23a1 containing the miR-298-5p binding site was cloned into the pmirGLO vector (Ribio, Guangzhou, China), designated as circLyst-WT-Report or col23a1-WT-Report. Site-directed mutation of circDcbld1 or the 3’ UTR of col23a1 was performed by the Quick Mutation site-directed mutagenesis kit (Beyotime Biotechnology, Shanghai, China). The mutated sequences were also cloned into the pmirGLO vector and named as circLyst-Mut-Report or col23a1-Mut-Report. The PCR primer sequences for Col23a1-WT, Col23a1-mutation (MUT), Circ2465-WT and Circ2465-MUT were shown in Table S7. HEK-293 T cells were co-transfected with the reporter plasmid or the mutated vectors with miR-298-5p mimics or negative control (NC). After 48 h, firefly and renilla luciferase activities were measured using the Dual-luciferase reporter assay system (Promega, USA).

### Bioinformatics analysis

MiRanda (http://www.microrna.org), TargetScan (http://www.targetscan.org) and RNAHybrid (https://bio.tools/rnahybrid) were used to predict the target miRNAs of circLyst. The miRNAs were ranked based on the potential binding affinity to circLyst. The target genes of the miRNA were also predicted and annotated by miRanda and TargetScan.

### Statistical analysis

All statistical analyses were conducted using GraphPad Prism 8 (GraphPad Software, San Diego, CA, USA). The data were reported as mean ± standard deviation (mean ± SD), with each experiment repeated at least three biological replicates. Normality of the data was assessed using the Shapiro–Wilk test. If the *P*-value from the Shapiro–Wilk test is greater than 0.05, the data is normally distributed, and parametric tests can be used. For comparisons between two groups, a paired t-test was employed for paired or matched data, while an F-test was conducted for unpaired experimental groups to assess variance equality. An unpaired t-test was used for data with equal variances, and an unpaired t-test with Welch's correction was employed for data with unequal variances. For comparisons among three or more groups, repeated measures one-way ANOVA was used followed by Tukey's post-hoc test for measurements on the same subjects across different conditions or time points. The Brown-Forsythe test assessed variance equality among unpaired experimental groups. If variances are equal, perform one-way ANOVA followed by Tukey’s test for comparing group means. If variances are unequal, conduct Brown-Forsythe and Welch ANOVA tests to adjust for uneven variances followed by Dunnett's T3 multiple comparisons test. In correlation analysis, the normality of the data was assessed, and Pearson's correlation coefficient (r) was used for normally distributed data, while Spearman's rank correlation coefficient (ρ) was employed for non-normally distributed data. The Mann–Whitney test was used for comparisons between two groups. Statistically significant differences were considered at *p* < 0.05, with *, **, and *** representing *p* values < 0.05, 0.01, and 0.001, respectively.

## Supplementary Information

Supplementary Material 1.

## Data Availability

Upon publication, the data, analytic methods, and study materials will be made openly available to other researchers. All the data generated or analysed during this study are included in this article and its supplementary information files. The raw sequencing data have been submitted to the NCBI Gene Expression Omnibus (GEO) and are available under accession number GSE334848 (https://www.ncbi.nlm.nih.gov/geo/query/acc.cgi?acc=GSE334848). This dataset is currently private and will be publicly released on June 08, 2027. Prior to public release, the data are available from the corresponding author upon reasonable request.
