# Supplementary material for: Mechanotransduction through oxidative stress upregulates circLyst to drive vascular remodeling in hypertensive humans and rodents
Source: Mol Biomed. 2026 Oct 13;7:209. doi: 10.1186/s43556-026-00593-3 (PMC13661865; doi:10.1186/s43556-026-00593-3)
Supplement: Supplementary file 1 [file 43556_2026_593_MOESM1_ESM.docx]

**Mechanotransduction Through Oxidative Stress Upregulates circLyst to Drive Vascular Remodeling in Hypertensive Humans and Rodents**

Juanjuan Tan^1,2†^, Ph.D., Weiyi Hu^2†^ Ph.D., Li Shen^3†^, M.D., Biyang Luo^2†^, M.D., Huajin Liu^4^, M.D., Feng Li^3^, B.S., Hui Qin^3^, M.D., Yuanwang Yu^1^, Ph.D., Haifang Wang^1^, Ph.D., Jing Dong^1^, M.D., Qi Zhang^1^, Ph.D., Hong Zhang^1^, Ph.D., Fan Lv^5^, M.D., Ph.D., Yingxin Qi^2^, M.D., Ph.D., Kai Wang^7*^, Ph.D., Yi Yang^6*^, M.D., Zhiqiang Yan^4*^, M.D., Ph.D., Kai Huang^2, 8*^

1. Shaanxi University of Chinese Medicine, Xianyang ,712046，China.

2. Institute of Mechanobiology & Medical Engineering, School of Life Sciences & Biotechnology, Shanghai Jiao Tong University, 800 Dongchuan Road, Minhang, 200240, Shanghai, China.

3. The Third Hospital of Changha (Changsha Hospital Affiliated to Hunan University), Changsha City, Hunan Province, 410015, China.

4. Shanghai Jiao Tong University affiliated sixth people's hospital south campus, Shanghai, China.

5. Department of Pediatric Surgery, Xinhua Hospital, Shanghai Jiao Tong University School of Medicine, 1665 Kongjiang Road, Yangpu, Shanghai, 200092, China.

6. Department of Cardiovascular Surgery, Ruijin Hospital, Shanghai Jiao Tong University School of Medicine, 200025, Shanghai, China.

7. The Key Laboratory of Bioactive Materials, Ministry of Education, College of Life Sciences, Nankai University, Tianjin, 300071, China.

8. Key Laboratory for the Genetics of Developmental and Neuropsychiatric Disorders, Ministry of Education, Shanghai Jiao Tong University, 800 Dongchuan Road, Minhang, 200240, Shanghai, China.

^†^These authors contribute equally to this work

^*^**Corresponding author:**

Kai Wang, 013053@nankai.edu.cn; Yi Yang, yy12181@rjh.com.cn; Zhiqiang Yan, zqyan@sjtu.edu.cn; or Kai Huang, huang_kai@sjtu.edu.cn.

**Supplementary Materials and Methods**

**Cell culture**

According to others and our previous studies[1-7], primary VSMCs and MSMCs were harvested from the thoracic aortas and mesenteric arterioles of healthy male 5-8 weeks old Sprague-Dawley rats by explant culture. Primary VSMCs were grown in DMEM (Thermo Fisher Scientific, USA) containing 10% fetal bovine serum (FBS) (Gibco) at 37 °C with 5% CO_2_. Primary MSMCs were grown in rat mesenteric smooth muscle cell complete medium (CM-R248, Procellsystem, China). The purity of the SMCs was confirmed using the α-SMA antibody (Cell Signaling Technology, Danvers, MA, USA). SMCs from passages 3 to 7 were used for all experiments.

Immortalized human aortic smooth muscle cells (Immortalized human ASMCs) were ordered from QuiCell (Shanghai, China) and cultured in smooth muscle cell culture medium (QuiCell-Pri-8006, Shanghai, China). Immortalized human ASMCs from passages 4 to 10 were used for all experiments. Renal artery specimens were collected from patients who underwent kidney tumor removal surgery at Shanghai Xinhua Hospital, and primary human ASMCs were isolated from renal arteries thereafter (approval ID: XHEC-D-2024-002). Primary human ASMCs were grown in DMEM (Gibco, New Zealand) containing 20% fetal bovine serum (FBS) (Sigma-Aldrich, Australia) at 37 °C with 5% CO_2_. Primary human ASMCs from passages 3 to 7 were used for all experiments.

All of the SMCs were starved with serum-free medium or DMEM containing 1% serum for 24 hours to arrest growth and synchronize the cells as we did previously[1-6, 8, 9] before subjecting to cyclic stretch or siRNA transfection[10].

**Dihydroethidium detection of superoxide production**

As we previously described[11, 12]，the fluorescent dye dihydroethidium (DHE) was used to detect total superoxide production in the aortas in situ and in the rat VSMCs. Arteries were harvested from all experimental rats at the end of the treatment protocol, and rings of the aortas were embedded in OCT compound at -80°C before use. Six μm frozen sections were cut using a cryostat (Leica, CM3050S) and mounted on glass slides. Slides were then rinsed briefly in Krebs/HEPES buffer to remove OCT compound. VSMCs were subjected to cyclic stretch as described above. Tissue sections and VSMCs were incubated with DHE (2 µmol/L) in a lightproof humidified container at 37°C for 30 min. Slides and cells were washed with Krebs/HEPES buffer 3 times, mounted using antifade mounting medium with DAPI (P0131, Beyotime Biotechnology, Shanghai, China), and imaged with a fluorescence microscope (Olympus IX81).

**Western blot**

Western blotting was performed following standard protocols. Briefly, Protein extract was electrophoresed on 10% SDS-PAGE precast gels (Invitrogen, USA). Proteins were detected using the following primary antibodies: rabbit anti-col23a1(1:500; Proteintech), rabbit anti-NOX1 (1:1,000; NBP1-31546, NOVUS biologicals), rabbit anti-NOX2 (1:1,000; NBP2-41291 NOVUS biologicals), rabbit anti-NOX4 (1:1,000; NB110-58849, NOVUS biologicals), rabbit anti- Calponin (1:1,000; GB11954-50, Servicebio), rabbit anti-α-actinin (1:1,000; GB111556-50, Servicebio), rabbit anti-SM22α (1:1,000; GB11366-50, Servicebio), abbit anti-β-actin (1:2000, 20536-1-AP, Proteintech) and mouse anti-GAPDH (1:2,000; Proteintech). The next day, membranes were incubated with HRP-conjugated secondary antibodies (1:2,000; Cell Signaling Technology) for 1 hr at room temperature. Blots were detected by an ECL kit (Beyotime) and quantified with Quantity One software (Bio-Rad).

**Cell proliferation assay**

Cell proliferation was analyzed using CCK-8 (Dojindo Molecular Technologies, Japan) and EdU (Ribio, Guangzhou, China) assay according to the instructions of manufacturer. The cell proliferation of each group was calculated using absorbance of treatment and control groups by CCK-8 assay. For EdU assay, 50 μM EdU was added to the culture media for 2 h, and proliferating cells were labeled as red and counted under a fluorescence microscope (Olympus TH-4-200).

**Cell migration assay**

Transwell system (Boyden chamber assay) and wound-healing assay were used to detect the cell migration. In the wound healing assay, VSMCs were seeded onto the 6-well culture plate with DMEM containing 1% serum for 24 h, reaching a 70%–80% confluence. A sterilized 200-μl pipette tip was used to gently and stably scratch in the center of the cell monolayer to create straight lines. The gap of the cell monolayer was photographed under a microscope (Leica) at 0 h, 12 h and 24 h at the same areas. In the transwell assay, 2×10^4^ cells were seeded in the upper Transwell chamber (8 mm pore size, BD) with 100 μl serum-free DMEM in a 24-well plate. 600 μl of DMEM with 20% FBS was placed in the bottom chamber. After 24 h, the migrated cells on the bottom of the membrane were fixed by 4% paraformaldehyde and stained by 0.1% crystal violet. And then, the cells were observed under a microscope (Olympus CKX41). Cells in random five 400X fields were counted to calculate the migration cell number.

**Immunofluorescence staining**

For immunofluorescence staining, tissue sections were fixed in 4% paraformaldehyde (PFA) for 10 min, permeabilized with 0.3% Triton X-100 for 20 min, and blocked with 10% goat serum for 1 h at room temperature. Subsequently, samples were incubated overnight at 4°C with the following primary antibodies: anti-α-SMA (1:200; Proteintech, 67735-1-Ig) and anti-Col23a1 (1:200; Proteintech, 14337-1-AP). After wash three times with PBS, sections were incubated with fluorophore-conjugated secondary antibodies for 1 h at room temperature, followed by nuclear counterstaining with DAPI (30 min). Images were acquired using a confocal microscope (Fluoview 1000, Olympus).

**Histological analysis**

At the end of the experiments, rats were euthanized with CO_2_. The aortas were rapidly removed, rinsed with ice cold Krebs/HEPES buffer, and cleaned of connective tissue and fat for histological analysis. The paraffin blocks were sectioned (6 μm), the staining procedures were conducted according to the commercially available protocol of H&E staining kit (C0105M, Beyotime, Shanghai, China) and Masson's Trichrome Staining Kit (C0189M, Beyotime, Shanghai, China).

**References**

1.	Tan J, Xie Y, Yao A, Qin Y, Li L, Shen L, et al. Long noncoding rna-dependent regulation of vascular smooth muscle cell proliferation and migration in hypertension. The international journal of biochemistry & cell biology. 2020;118:105653. <https://doi.org/10.1016/j.biocel.2019.105653>.

2.	Yang YC, Wang XD, Huang K, Wang L, Jiang ZL, Qi YX. Temporal phosphoproteomics to investigate the mechanotransduction of vascular smooth muscle cells in response to cyclic stretch. J Biomech. 2014;47(15):3622-3629. <https://doi.org/10.1016/j.jbiomech.2014.10.008>.

3.	Huang K, Yan ZQ, Zhao D, Chen SG, Gao LZ, Zhang P, et al. Sirt1 and foxo mediate contractile differentiation of vascular smooth muscle cells under cyclic stretch. Cell Physiol Biochem. 2015;37(5):1817-1829. <https://doi.org/10.1159/000438544>.

4.	Qi YX, Yao QP, Huang K, Shi Q, Zhang P, Wang GL, et al. Nuclear envelope proteins modulate proliferation of vascular smooth muscle cells during cyclic stretch application. Proc Natl Acad Sci U S A. 2016;113(19):5293-5298. <https://doi.org/10.1073/pnas.1604569113>.

5.	Bao H, Li HP, Shi Q, Huang K, Chen XH, Chen YX, et al. Lamin a/c negatively regulated by mir-124-3p modulates apoptosis of vascular smooth muscle cells during cyclic stretch application in rats. Acta Physiol (Oxf). 2020;228(3):e13374. <https://doi.org/10.1111/apha.13374>.

6.	Ren H, Chen J, Huang K, Qi YX. Platelet-derived microvesicles drive vascular smooth muscle cell migration via forming podosomes and promoting matrix metalloproteinase-9 activity. Mechanobiology in Medicine. 2023;1(1):100003.

7.	Dubey RK, Overbeck HW. Culture of rat mesenteric arteriolar smooth muscle cells: Effects of platelet-derived growth factor, angiotensin, and nitric oxide on growth. Cell Tissue Res. 1994;275(1):133-141. <https://doi.org/10.1007/BF00305381>.

8.	Huang K, Bao H, Yan ZQ, Wang L, Zhang P, Yao QP, et al. Microrna-33 protects against neointimal hyperplasia induced by arterial mechanical stretch in the grafted vein. Cardiovasc Res. 2017;113(5):488-497. <https://doi.org/10.1093/cvr/cvw257>.

9.	Chen Y, Bao M, Liu JT, Bao H, Zhang SM, Lou Y, et al. Defective autophagy triggered by arterial cyclic stretch promotes neointimal hyperplasia in vein grafts via the p62/nrf2/slc7a11 signaling pathway. J Mol Cell Cardiol. 2022;173:101-114. <https://doi.org/10.1016/j.yjmcc.2022.10.001>.

10.	Tan J, Yang L, Liu C, Yan Z. Microrna-26a targets mapk6 to inhibit smooth muscle cell proliferation and vein graft neointimal hyperplasia. Sci Rep. 2017;7:46602. <https://doi.org/10.1038/srep46602>.

11.	Huang K, Narumi T, Zhang Y, Li Q, Murugesan P, Wu Y, et al. Targeting microrna-192-5p, a downstream effector of noxs (nadph oxidases), reverses endothelial dhfr (dihydrofolate reductase) deficiency to attenuate abdominal aortic aneurysm formation. Hypertension. 2021;78(2):282-293. <https://doi.org/10.1161/HYPERTENSIONAHA.120.15070>.

12.	Jiang J, Huang K, Xu S, Garcia JGN, Wang C, Cai H. Targeting nox4 alleviates sepsis-induced acute lung injury via attenuation of redox-sensitive activation of camkii/erk1/2/mlck and endothelial cell barrier dysfunction. Redox biology. 2020;36:101638. <https://doi.org/10.1016/j.redox.2020.101638>.

**Figure S1. The expression of miR-298-5p, circLyst and col23a1 expression after miR-298-5p mimics transfection.** Transfection with miR-298-5p mimics at concentrations of 50, 100, and 200 μM in VSMCs. a The expression of miR-298-5p after transfection with miR-298-5p mimics at concentrations of 50, 100, and 200 μM in VSMCs. Data were presented as mean ± SD. n = 3, Two-way ANOVA followed by Bonferroni-corrected Welch T tests: ***p<0.0001 (50μM), ***p<0.0001 (100μM), ***p<0.0001 (200μM), miR-298-5p mimics group versus NC group. b The expression of circLyst after transfection with miR-298-5p mimics at concentrations of 50, 100, and 200 μM in VSMCs. Data were presented as mean ± SD. n = 3, Two-way ANOVA followed by Bonferroni-corrected Welch T tests: *p=0.039 (50μM), ***p=0.0003 (100μM), ***p=0.0005 (200μM), miR-298-5p mimics group versus NC group. c The expression of col23a1 after transfection with miR-298-5p mimics at concentrations of 50, 100, and 200 μM in VSMCs. n = 3, Two-way ANOVA followed by Bonferroni-corrected Welch T tests: **p=0.0034 (100μM), ***p=0.0002 (200μM), miR-298-5p mimics group versus NC group.

**Figure S2. Col23a1 was elevated both in human and rodent under cyclic stretch.** a Rat VSMCs was harvested and subjected to 5% or 15% cyclic stretch respectively, col23a1 protein expression was detected in VSMCs under cyclic stretch for 6 hours. n=3, Welch T test: **p=0.0059, 15% cyclic stretch versus 5% cyclic stretch. b Immortalized human ASMCs were subjected to 5% or 15% cyclic stretch respectively, col23a1 protein expression was detected in ASMCs under cyclic stretch for 6 hours. n=4, Welch T test: *p=0.0241, 15% cyclic stretch versus 5% cyclic stretch. c Immortalized human ASMCs were subjected to 5% or 15% cyclic stretch respectively, col23a1 protein expression was detected in ASMCs under cyclic stretch for 24 hours. n=4, Welch T test: *p=0.0187, 15% cyclic stretch versus 5% cyclic stretch. d Primary human ASMCs were subjected to 5% or 15% cyclic stretch respectively, col23a1 protein expression was detected in ASMCs under cyclic stretch for 6 hours. n=4, Welch T test: ***p=0.0002, 15% cyclic stretch versus 5% cyclic stretch. e Primary human ASMCs were subjected to 5% or 15% cyclic stretch respectively, col23a1 protein expression was detected in ASMCs under cyclic stretch for 24 hours. n=4, Welch T test: *p=0.0127, 15% cyclic stretch versus 5% cyclic stretch. f Rat MSMCs was subjected to 5% or 15% cyclic stretch respectively. Col23a1, α-actin, calponin and SM-22 were detected in MSMCs under cyclic stretch for 24 hours. n=4, Welch T test with Holm-Šidák correction for four independent comparisons (familywise α=0.05): *p=0.0181(Col23a1), **p=0.0087(α-actin), *p=0.0338(SM-22), 15% cyclic stretch versus 5% cyclic stretch. g Rat MSMCs was co-transfected with miR-298-5p inhibitors and siRNA-circLyst. Col23a1, α-actin, calponin and SM-22 were detected in MSMCs after co-transfection. n=3, Welch T test with Holm-Šidák correction for four independent comparisons (familywise α=0.05): **p=0.0054(Col23a1), **p=0.0048(α-actin), *p=0.0357(calponin), *p=0.0166(SM-22), 15% cyclic stretch versus 5% cyclic stretch.

**Table supplement 1. circRNA_2465 sequence**

Sequence (5’-3’)

ACCCTTCTTGAAGGTTGCTGTGGTGAAGAAGTTATCTTCGTCAATGAGCATGGAGAGTTCAAGTTGGATGTTGAATCTCATGCTATAATCCAAGATGTTAAACTGTTGCAGGAACTGTTGCTTGACTGGAAGATATGGAATAAGGCAGAGCAAGGCGTGTGGGAGACTCTGCTGGCAGCTCTGGAAGTCCTCATCCGGGTAGAGCACCAGCAGCAGCAGTTTAATATTACGCAGTTGCTGAACGCCCGTGTCGTTCACCACTTCCTACTGACCTGTCAGGTTTTACAGGAACACAGAGAGGGGCAGCTTACATCAATGCCCCGAGAAGTTTGTAGATCGTTTGTGAAAATTATTGCAGAAGTCCTTGGTTCTCCTCCAGACTTGGAATTATTGTCAGTTATCTTCAATTTCCTGTTAGCAGTACACCCTCCTACTAATACTTATGTTTGTCACAACCCTGCAAACTTCTACTTCTCTTTGCACATAGATGGCAAGATCTTTCATGAGAAAGTGCGGTCAATCATGTACCTGAGGCATTCTAGCAGTGGAGGGCAATCCTTTCCCAGCCCTGGGTTTATGGTAATAAGCCCGTCTGCCTTTACTGCATCTCCTCCTGAAGGAACCAGTTCTTCCAATATTGTTCCACAGAGGATGGCTGCCCAGATGGTACGATCTAGAAGTCTACCAGCATTTCCTACTTATTCACCACTAATGCAAGCACAAAAACTGGCGGGAAGTTTGGTCTTTAGTGTTGACAAGTTACAAAATATTGCAGATGCCAGCCCAGAGAAACGGAATCCTTTAGGCAGTCCCTACACATTGAAAAAAAGCAAAGAGGAAGCATTCATTAGTAGCTGTGAGTCTGCAAAAACTGTTTGTGAAATGGAGGCATTTCTTGCAGCCCAGGCCTCTGCCAATGGGGTCTCCAGAGGTTCACTGAGGTTTCCCATGGCCAGAATAGATCACAAAGACTTGGGGACTGAGCACAGATCAGATGATGACAGTCCTGGGGATGAGTCTTATCCACGTCGACCTGATAACCTCAAGGGACTGGCCTCATTCCAGCGAAGCCACAGCACTATTGCGAGCCTGGACTTGCATTTCCCTAATGGATCTGCAGTTGCTAGCAGATGGCCGAGTCTTGTTGATAGGAATGCTGATGATTGGGAAAACTTTACCTTTTCTTATGAGGCAAACTACAACCGAGCTACAAGCACCCACAGTGCCATTGAAGACTGTTTGATACCTATCTGCTGTGGATTTTATGAACTCCTAAGTGGGGTTCTTCTTGTTCTGCCTGATGCTATGCTTGAAGATGTGATGGACAGGATTATTCAAGCAGATATTCTTCTAGTCCTTGTTAACCACCCATCACCTGCTATCCAGCAAGGAGTAATTAAACTGTTACAAGCATACATTAATAGAGCATCAAAGGAACAAAAGGACAAGTTTCTGAAGAACCGTGGCTTTTCCTTACTAGCCAACCAGTTGTATCTTCACAGGGGAACTCAGGAGTTGTTGGAGTGCTTCATTGAAATGTTCTTTGGTCGGCCATTTGGCCTGGATGAAGAATTTGATCTGGATGAAATGAAACACATGGAACTGTTCCAGAAGTGGTCTGTCATTCCAATTCTGGGACTAATAGAGACCTCTCTCTATGACAATATACTCTTGCACAATTCTCTTTTACTTCTTCTGCAAGTTTTAAACTCTTGTTCCAAGGTAGCAGACATGCTGCTAGACAATGGACTGCTCTATGTGTTATGTAATACAGTAGCAGCCCTGAATGGATTAGAAAAGAACATTCCTTTGAATGAATTCAGATTGCTCGCATGCGATATACAGCAACTTTTCATAGCGGTTACAATCCACGCTTGCAGTTCTTCGGGCTCACAGTATTTTAGAGTTATCGAAGACCTCATTGTACTTCTTGGATATCTTCATAATAGCAAAAATAAGAGGACACAAAATATGGCTTTGGCCCTCCAGCTTAAAGTTCTCCAGGCTGCTTTGGAATTTATAAGGAGCACAGCCAATCACGACTCTGAAAGTCCCTTAGATGCAGTGCAGTCGCCTTCTGCCTACCACCATTCAGTGCCTCAGAAGAGGAGAAGCATTGCTGGTCCTCGCAAATTCCCCCTGGCTCAGACAGAGTCCCTCCTGATGAAGATGCGCTCGGTGGCCAGTGATGAGCTGCACTCTATGATGCAGAGGAGGATGAGCCAGGAGCACCCCAGCCAGGCCTCAGAGGCAGAGCTCGCCCAGCGGCTGCAGAGGCTCATCATCTTAGCTGTGAACAGGATCATTTACCAAGAGTTGAATTCAGATATTATTGACATTTTGAGAACTCCAGAAAATACATCCCAAAGCAAGACCTCAGTGTCCCAGACTGAAACTTCTGAAGAAGATGTGCATTATGAGCAACCTTCTGTATCCAATCCATTCCAAAAAGAAATGTTCACATATCTGTTGGATGGTTTCAAAATATCTATTGGTTCAAGTAAAACTGGCACTTCTAAGATATTTTGTCACCAACCCACACTGTACAAGAAAGGAAGCAGATACTTGAGATAGTCCATGAACCAGCTCATCAGGATATACTTCGTGACTGTCTCAGCCCCTCCCCACAA

**Table supplement 2. Individual information of hypertensive patient and the respective control.**

| **Group** | **Gender** | **Age (years)** | **BMI (kg/m2)** | **Blood pressure (mmHg)** |
| --- | --- | --- | --- | --- |
| Control 1 | male | 47 | 22 | 110/70 |
| Control 2 | male | 43 | 24 | 125/78 |
| Control 3 | male | 46 | 31 | 130/72 |
| Control 4 | male | 40 | 22 | 115/72 |
| Control 5 | female | 32 | 24 | 105/72 |
| Control 6 | female | 33 | 23.1 | 110/60 |
| Control 7 | female | 41 | 21 | 124/78 |
| Control 8 | male | 22 | 23 | 115/68 |
| Control 9 | male | 23 | 23.4 | 120/72 |
| Control 10 | male | 20 | 22 | 102/70 |
| Control 11 | female | 41 | 22.5 | 105/61 |
| Control 12 | female | 37 | 23.3 | 120/68 |
| Control 13 | female | 43 | 21 | 100/70 |
| Control 14 | female | 38 | 23.1 | 90/60 |
| Control 15 | female | 24 | 23.5 | 107/80 |
| Control 16 | male | 25 | 24 | 116/75 |
| Control 17 | male | 22 | 23.4 | 109/74 |
| Hypertension 1 | male | 67 | 29.3 | 180/110 |
| Hypertension 2 | female | 82 | 24.8 | 180/90 |
| Hypertension 3 | male | 61 | 25 | 148/98 |
| Hypertension 4 | male | 48 | 21.5 | 160/110 |
| Hypertension 5 | male | 74 | 27.5 | 149/92 |
| Hypertension 6 | male | 39 | 27.6 | 170/100 |
| Hypertension 7 | female | 74 | 25.4 | 179/95 |
| Hypertension 8 | male | 77 | 23.5 | 151/74 |
| Hypertension 9 | female | 54 | 24.8 | 170/100 |
| Hypertension 10 | male | 67 | 22.3 | 190/108 |
| Hypertension 11 | male | 52 | 26 | 180/100 |

**Table supplement 3. PCR primer sequences for circRNAs and mRNA**

|  | **Primer sequence (5’-3’)** | **Product (bp)** |
| --- | --- | --- |
| circRNA_2465 | Forward: TGTCACCAACCCACACTG | 241 |
|  | Reverse: CGCCTTGCTCTGCCTTAT |  |
| circRNA_4907 | Forward: GCCACAGTAACCATCTTGGA | 134 |
|  | Reverse: CAAATACGAAACTTCTGGAGGC |  |
| circRNA_6043 | Forward: AGACACTGCTGCTGAAGGTT | 114 |
|  | Reverse: GAGATGCCCGATCGCAATTC |  |
| circRNA_1280 | Forward: GCGAGCGCATCTTCTTACAC | 298 |
|  | Reverse: GCCCCTCTTGGTCAGCTTAG |  |
| circRNA_1400 | Forward: AGCAGCTGAGTATTGCGGAG | 173 |
|  | Reverse: GACTGCTTAGCGAGGAGGAC |  |
| circRNA_1725 | Forward: CTCCTACCGTAATGCCCTGC | 292 |
|  | Reverse: TCATTCTCTCGCTGCTCGTT |  |
| Lyst | Forward: GTTGTGCCTGGGTGCCTGAC | 90 |
|  | Reverse: TGCCGCTTGTGAACCTGTTGG |  |

**Table supplement 4. siRNA sequences for circRNA-2465**

|  | **Sequence (5’-3’)** |
| --- | --- |
| CircRNA-2465-siRNA-1 | Sense：CCCUCCCCACAAACCCUUCTT |
|  | Antisense：GAAGGGUUUGUGGGGAGGGTT |
| CircRNA-2465-siRNA-2 | Sense：CCCACAAACCCUUCUUGAATT |
|  | Antisense：UUCAAGAAGGGUUUGUGGGTT |
| siRNA Negative control (NC) | Sense:UUCUCCGAACGUGUCACGUTT |
|  | Antisense:ACGUGACACGUUCGGAGAATT |

**Table supplement 5. The sequences of miR-298-5p mimics and inhibitors**

|  | **Sequence (5’-3’)** |
| --- | --- |
| miR-298-5p mimics | Sense：GGCAGAGGAGGGCUGUUCUUCCC |
|  | Antisense：GAAGAACAGCCCUCCUCUGCCUU |
| Negative control (NC) | Sense：UUCUCCGAACGUGUCACGUTT |
|  | Antisense：ACGUGACACGUUCGGAGAATT |
| miR-298-5p inhibitors | GGGAAGAACAGCCCUCCUCUGCC |
| microRNA inhibitor NC | CAGUACUUUUGUGUAGUACAA |

**Table supplement 6. The sequences of circ_2465 shRNA**

| **circ_2465 shRNA** | |
| --- | --- |
| LV3-H1/GFP&Puro-circ_2465-shRNA | CCCTCCCCACAAACCCTTC |
| LV3-shNC | GTTCTCCGAACGTGTCACGT |

**Table supplement 7.** **PCR primer sequences for Col23a1-WT, Col23a1-mutation (MUT), Circ2465-WT and Circ2465-MUT**

|  | **Sequence (5’-3’)** |
| --- | --- |
| Col23a1-WT forward primer | AACGAGCTCGCTAGCCTCGAGCTGTGGATACCTCACTGGCA |
| Col23a1-WT reverse primer | CCTGCAGGTCGACTCTAGAATATGCAGAGGGATGTGAGGCC |
| Col23a1-mutation (MUT) forward primer | AACGAGCTCGCTAGCCTCGAGCTGTGGATACCTCACTGGCA |
| Col23a1-MUT reverse primer | CCTGCAGGTCGACTCTAGAATATTAGTCCAGATGTGAGGCC |
| Circ2465-WT forward primer | AACGAGCTCGCTAGCCTCGAGAAGAAAGTGGTTATACCCCTGC |
| Circ2465-WT reverse primer | CCTGCAGGTCGACTCTAGACTGTAAAGAAGTTCTTCTGG |
| Circ2465-MUT forward primer | AACGAGCTCGCTAGCCTCGAGGCTCGAGTGGTTATACCCACTA |
| Circ2465-MUT reverse primer | CCTGCAGGTCGACTCTAGACTGTAAAGAAGTTCTTCTGG |
